# Synthesis and Evaluation of Non‐Hydrolyzable Phospho‐Lysine Peptide Mimics

**DOI:** 10.1002/chem.202003947

**Published:** 2020-12-07

**Authors:** Anett Hauser, Eleftheria Poulou, Fabian Müller, Peter Schmieder, Christian P. R. Hackenberger

**Affiliations:** ^1^ Leibniz-Forschungsinstitut für Molekulare Pharmakologie (FMP) Robert-Rössle-Strasse 10 13125 Berlin Germany; ^2^ Department of Chemistry Humboldt-Universität zu Berlin Brook-Taylor-Strasse 2 12489 Berlin Germany

**Keywords:** amino acids, chemoselectivity, phosphorylation, post-translational modification, solid-phase peptide synthesis analogues

## Abstract

The intrinsic lability of the phosphoramidate P−N bond in phosphorylated histidine (pHis), arginine (pHis) and lysine (pLys) residues is a significant challenge for the investigation of these post‐translational modifications (PTMs), which gained attention rather recently. While stable mimics of pHis and pArg have contributed to study protein substrate interactions or to generate antibodies for enrichment as well as detection, no such analogue has been reported yet for pLys. This work reports the synthesis and evaluation of two pLys mimics, a phosphonate and a phosphate derivative, which can easily be incorporated into peptides using standard fluorenyl‐methyloxycarbonyl‐ (Fmoc‐)based solid‐phase peptide synthesis (SPPS). In order to compare the biophysical properties of natural pLys with our synthetic mimics, the p*K*_a_ values of pLys and analogues were determined in titration experiments applying nuclear magnetic resonance (NMR) spectroscopy in small model peptides. These results were used to compute electrostatic potential (ESP) surfaces obtained after molecular geometry optimization. These findings indicate the potential of the designed non‐hydrolyzable, phosphonate‐based mimic for pLys in various proteomic approaches.

## Graphical abstract

**A stable mimic**: This work introduces a phosphonate amino acid as a stable non‐hydrolyzable mimic of phospho‐lysine (pLys), which can be readily incorporated into peptides by Fmoc‐based solid‐phase peptide synthesis.

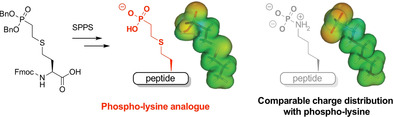

## Introduction

Post‐translational phosphorylation of proteins occurs on almost all nucleophilic amino acid side chains.^[1]^ Vast efforts, especially in the area of phosphoproteomics, are undertaken to identify new phosphorylation sites and advance the understanding of underlying mechanisms of this most important post‐translational modification. For the highly abundant, acid‐stable phosphate esters of serine, threonine and tyrosine (pSer, pThr, pTyr), phospho‐specific enrichment and mass spectrometric‐ (MS‐)based phosphoproteomic methods yielded thousands of phosphorylation sites.^[2]^ In contrast, acid‐labile phosphorylations are less explored and the chemical and temperature‐related intrinsic lability is particularly challenging. These specific experimental requirements have to be met by adjustment of existing cell extraction, enrichment protocols and MS techniques. Recent reports on His and Arg phosphorylation demonstrate the successful improvements of phospho‐binding strategies including TiO_2_,^[3]^ immobilized metal affinity chromatography (IMAC),^[4]^ hydroxyapatite^[5]^ or strong anion exchange (SAX),^[6]^ despite the fact that virtually no discrimination between various phospho‐species can be achieved with these methods. Besides that, phospho‐amino acid‐specific protocols could be developed for pHis and pArg employing stable analogues of the endogenous phosphorylation. Such molecules were designed for the generation of antibodies (Abs)^[7]^ or to study interacting proteins.^[8]^ Among phosphoramidates, pLys is less studied and its role in the cellular context remains to be examined. Detected in vivo already in 1977, pLys has been reported as an acid‐labile PTM of histone H1^[9]^ and only preliminary details on interacting proteins have been published.^[10]^ Despite recent reports on incidental pLys identification with phospho‐specific enrichment techniques[^5^, ^6^] and an indirect proof for the phosphorylation event via derivatization,^[11]^ no pLys‐mimicking analogues suitable for Ab generation or binding partner examination were obtained so far.

**Figure 1:**
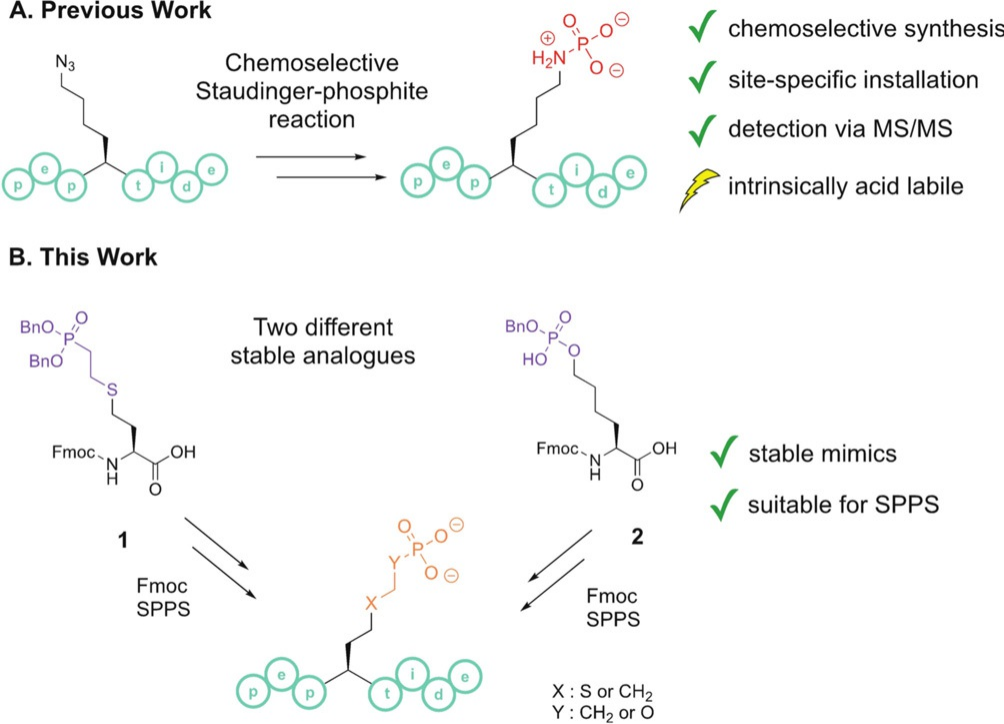
A. pLys peptides can be obtained in directly from solid support or after cleavage from resin in a two‐step protocol. B. pLys analogues **1** and **2** can be installed during SPPS to obtain acid‐stable phosphonates (X: S, Y: CH_2_) or phosphates (X: CH_2_, Y: O).

To enable the study of lysine phosphorylation, our group has recently developed two different synthetic methods for the site‐selective synthesis of pLys peptides. Such peptides can be obtained via the chemoselective Staudinger‐phosphite reaction^[12]^ delivering either a photo‐caged pLys in unprotected peptides^[13]^ or free phosphoramidates when combining a base‐cleavable solid support and base‐labile phosphoramidate protecting groups^[14]^ (Figure 1 A). With those model peptides in hand, the stability of pLys under various pH and temperature conditions as well as suitable tandem‐MS techniques were investigated.[^13^, ^15^]

To augment the box of chemical tools to study lysine phosphorylation, we now propose two different Fmoc‐protected amino acid building blocks as stable pLys mimics, namely phosphonate **1** and phosphate **2** (Figure 1 B).

In order to evaluate the ability of our proposed mimics to serve as pLys mimics, p*K*_a_ values of peptides containing either **1**, **2** or pLys were determined in NMR titration experiments and the electron densities visualized as electrostatic potential (ESP) maps. Our findings demonstrate that both building blocks show sufficient stability and can be incorporated into peptides by standard SPPS in contrast to a phosphoramidate building block, which would deliver the native pLys peptide.

Furthermore, the observed p*K*_a_ value of phosphonate **1** points towards the great potential of this analogue to be used as pLys surrogate mimic in future applications, such as Ab generation and substrate‐protein interaction studies.

## Results and Discussion

### Design of phospho‐lysine analogues

Phosphoramidates contain a high energy P−N bond (Δ*G*°=−10.3 to −14 kcal mol^−1^),^[16]^ which translates in a decreased stability at lower pH and higher temperatures. Presumably, pLys is prone to hydrolysis due to the protonation of the *ϵ*‐nitrogen (p*K*_a_ of *N*‐(*n‐*butyl) phosphoramidate is 9.9),^[17]^ even though this value has never been determined for pLys itself. Besides the unique charge distribution illustrated in Figure 1 A, the length of the alkyl side chain is the most characteristic property of pLys. Therefore, we envisioned two types of phosphorous derivatives to identify a suitable pLys mimic (Figure 1 B), which are both easily accessible: The first derivative **1** exhibits a non‐hydrolyzable phosphonate, which would be obtained via a conjugate addition of alkene‐phosphonates with homocysteine (hCys).^[18]^ The other analogue **2** is a phosphate monoester derived from 6‐hydroxynorleucine with an improved pH and temperature stability profile. As both building blocks were envisioned to be incorporated into peptides by Fmoc‐based SPPS and give free phosphonate or phosphate directly upon cleavage from the solid support, we determined acid‐labile benzyl (Bn) groups as suitable for side chain protection.

### Synthesis phospho‐lysine mimic building blocks for SPPS

**Scheme 1:**
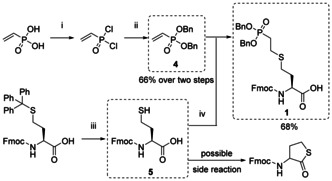
Synthesis of phosphonate mimic **1**. (i) (COCl)_2_, CH_2_Cl_2_, cat. DMF, 0 *°*C to rt., 19 h; (ii) BnOH, Et_3_N, THF, 0 *°*C to rt., 17 h; (iii) TFA/TIS/EDT/CH_2_Cl_2_ (50/2/0.1/47.9), 0 *°*C, 5 min; (iv) 50 mm NaHCO_3_/DMF (2/1), pH 8.5, 50 *°*C, 4 h; (Bn=benzyl, Ph=phenyl, Fmoc=fluorenylmethyloxycarbonyl, DMF=dimethylformamide, THF=tetrahydrofuran, TFA=trifluoroacetic acid, TIS=triisopropylsilane, EDT=1,2‐ethanedithiol).

The bis‐Bn‐ and Fmoc‐protected phosphonate building block **1** (Fmoc‐hCys(OBn)_2_)‐OH) was obtained as a thioether of Fmoc‐protected hCys (Scheme 1). First, vinylphosphonic acid was converted into the benzyl ester in two steps to give vinyl‐phosphonate **4**. Therefore, commercially available Fmoc‐hCys(Trt)‐OH was deprotected under acidic conditions using 50 % trifluoroacetic acid (TFA), 2 % triisopropylsilane (TIS) and 0.1 % ethanedithiol (EDT) in CH_2_Cl_2_. It became evident that short reaction times, cooling of the reaction mixture and immediate purification of the free thiol **5** were required to minimize cyclization and thiolactone formation. For example, running the deprotection for 30 minutes and purifying the reaction mixture after overnight EDT evaporation yielded approximately 50 % conversion to the thiolactone. Under optimized conditions, **5** was isolated in 80 % yield with 6 % thiolactone as side product. Conjugate addition of **4** to **5** at slightly basic pH 8.5 and elevated temperatures delivered compound **1** in 68 % yield.

**Scheme 2:**
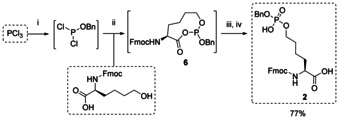
Synthesis of Fmoc‐Nle(OPO(OH)(OBn))‐OH **2**. (i) BnOH, THF, 0 *°*C, 5 min; (ii) 2,6‐lutidine, 0 *°*C to rt., 90 min; (iii) H_2_O, 0 *°*C, 5 min; (iv) NaBr, NaBrO_3_, 0 *°*C to rt., 72 h; (Bn=benzyl, Fmoc=fluorenylmethyloxycarbonyl, THF=tetrahydrofuran).

Bn‐ and Fmoc‐protected phosphate **2** could be obtained in a convenient one‐pot synthesis following a previously described protocol for Bn‐protected pSer, pThr and pTyr derivatives^[19]^ (Scheme 2). Starting from phosphorous trichloride, the first chloride atom was exchanged with benzyl alcohol. Subsequent reaction with commercially available Fmoc‐Nle(6‐OH)‐OH furnished the proposed, intermediate cyclic phosphite **6**, which was directly hydrolyzed and oxidized to yield the desired compound **2** in 77 % yield.

### Peptide synthesis with phospho‐lysine analogues

**Scheme 3:**
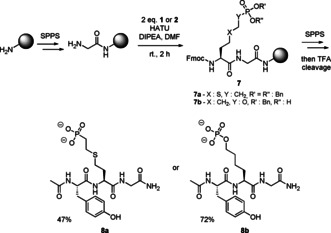
Fmoc‐based SPPS of peptides employing mimics **1** and **2**.

Next, we used both Fmoc‐protected building blocks **1** and **2** in the synthesis of tripeptides **8 a** and **8 b** (Scheme 3). To measure p*K*_a_ values for the phosphorous moiety in a peptidic system, we synthesized **8** with a C‐terminal amide, an N‐terminal acetyl group, a glycine and a UV‐active Tyr residue. **1** and **2** (2 equiv) were coupled using HATU ((1‐[Bis(dimethylamino)methylene]‐1*H*‐1,2,3‐triazolo[4,5‐b]pyridinium3‐oxide‐hexafluoro‐phosphate) to deliver peptides **7 a** and **7 b** on the solid support followed by standard Fmoc‐SPPS protocols. For the thioether‐containing peptide **8 a**, EDT and trimethylsilylbromide were added to the cleavage cocktail to avoid oxidation to the sulfoxide.^[20]^ Both peptides **8 a** and **8 b** were obtained in good isolated yields, which demonstrates the acid stability of these mimics as well as the general applicability of building blocks **1** and **2** in standard Fmoc‐based SPPS.

**Scheme 4:**
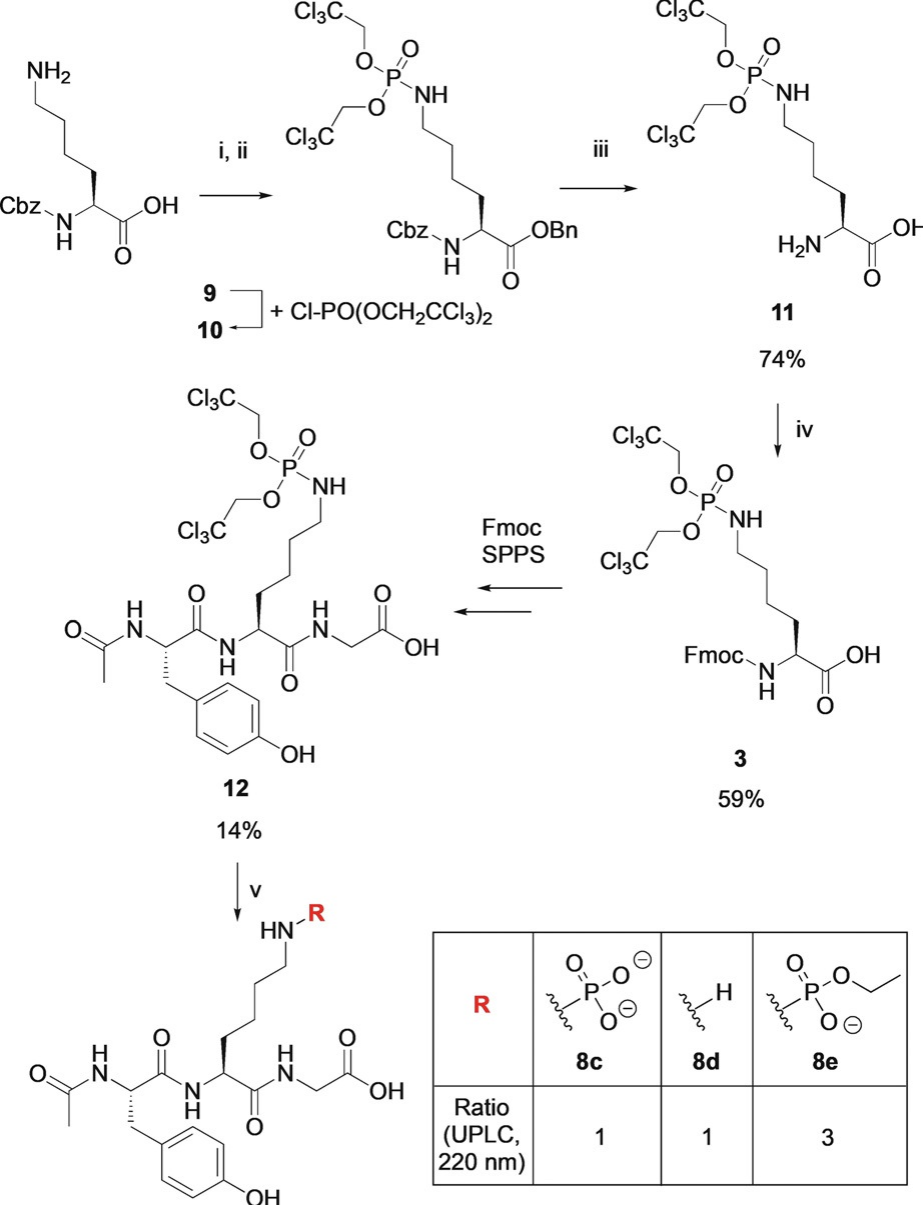
Synthesis of Fmoc‐Lys(NPO(OTc)_2_)‐OH **3** and Fmoc‐based SPPS employing monomer **3**. (i) SOCl_2_, BnOH neat, 0 °C to rt., 2 h; (ii) bis(2,2,2‐trichloroethyl) phosphorochloridate, Et_3_N, ACN, rt., 5 h; (iii) H_2_, Pd/C, AcOH/TFA/MeOH (5/5/90), rt., 1 h; (iv) Fmoc‐OSu, Et_3_N, H_2_O/ACN (1/1), rt., 4 h; v) H_2_, 10 % Pd/C, (NH_4_)_2_CO_3_ pH 8.0, EtOH, r.t, 2 h, (Bn=benzyl, Cbz=benzyloxycarbonyl, Fmoc=fluorenylmethyloxycarbonyl, TFA=trifluoroacetic acid, Su=succinimide).

Furthermore, to compare the p*K*_a_ values of **1**‐ or **2**‐containing peptides with pLys peptides, we also aimed to synthesize the corresponding pLys peptide **8 c** (^Ac^Tyr*pLys*Gly^CONH2^). In light of the successful Fmoc‐based SPPS of pArg peptides using a bis(2,2,2‐trichloroethyl)‐ (Tc‐)protected pArg building block,^[21]^ we wanted to test whether a corresponding pLys building block would be suitable for Fmoc‐based SPPS. We hypothesized that the electron‐withdrawing Tc‐protecting group would increase the stability of the P−N bond against TFA treatment required for peptide deprotection and cleavage. As shown in Scheme 4, the Tc‐protected pLys building block **3** was obtained starting from benzyloxycarbonyl‐ (Cbz‐)protected Lys, in which the acid was first converted into the benzyl ester **9** before the protected phosphoramidate **10** was formed via nucleophilic substitution with bis(2,2,2‐trichloroethyl) phosphorochloridate. Change of protecting groups to building block **3** was induced by hydrogenation, in which acidic conditions were required to not affect the Tc‐protecting group, followed by reaction with Fmoc‐succinimide (Fmoc‐OSu). Monomer **3** was obtained after purification on silica column in 59 % overall yield. Next, the building block **3** was tested toward its performance in SPPS analogous for building blocks **1** and **2** using HATU‐coupling conditions. While we were able to isolate the desired product **12**, we also observed substantial formation of side products (Figure S1). Furthermore, the deprotection of **12** to give the free phosphoramidate was only possible to a certain extent under the previously reported hydrogenation conditions despite considerable optimization efforts (Scheme 4).

While the desired peptide **8 c** was formed with maximum 20 % conversion and significant P−N bond cleavage to the lysine peptide **8 d** was observed, the major fraction of detected product was the mono‐ethyl‐protected intermediate **8 e**.

Because of these observations, we decided to synthesize peptide **8 c** following our chemoselective in solution protocol,^[13]^ which delivered peptide **8 c** in 45 % yield, significantly higher than the yield obtained with the Tc‐protected pLys approach (see SI, section 3.5). With the three peptides **8 a**–**c** in hand, we tested their enzymatic stability against the promiscuous alkaline phosphatase (ALP). The substrates **8 a**–**c** were tested for stability towards ALP under optimized conditions. Briefly, 1 mm substrate was dissolved in 50 mm Tris, 1 mm MgCl_2_, 1 mm ZnCl_2_, pH 8.2, at 30 °C and was incubated with 0.05 U ALP for 2 h. and the amount of released inorganic phosphate (P_i_) was determined via photometric read‐out. ALP hydrolyzed the phosphoramidate bond in **8 c** as expected, thereby demonstrating enzyme activity. Furthermore, treatment of **8 b** with ALP yielded in a P_i_ release, which indicated susceptibility of the phosphate analogue towards phosphatases. To our delight, the phosphonate analogue **8 a** remained intact when incubated with ALP, further verifying the stability of this derivative against hydrolysis (Figure S2).

### Charge distribution of phospho‐lysine and its mimics

**Figure 2:**
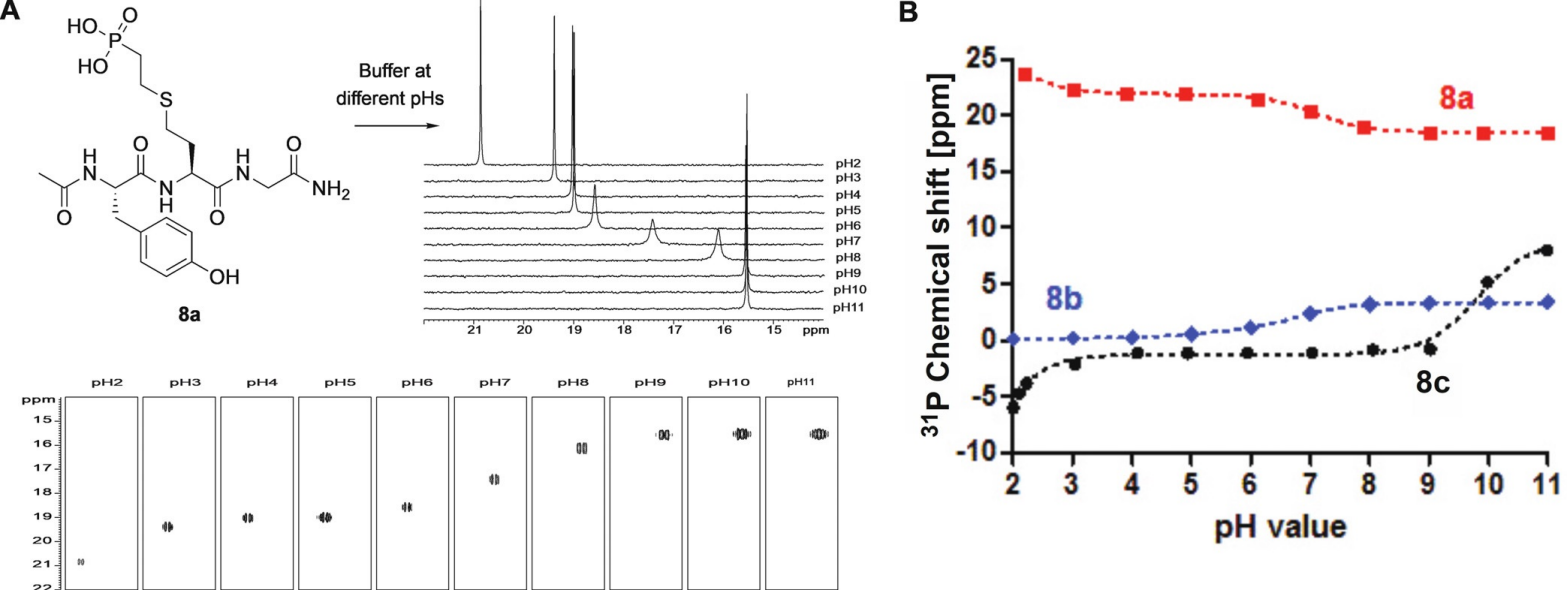
A. NMR titration experiments of peptide **8** **a**. Conditions: NMR spectra of a solution containing 1 mm peptide in 40 mm KCl (H_2_O+10 % D_2_O) were recorded at distinct pH values on a 600 MHz (^1^H frequency) spectrometer at 278 K. Shown are the overlay of 1D‐^31^P spectra at different pH values and extracted correlation signals between *ϵ*‐protons and phosphorous from ^1^H,^31^P‐HMBC experiments. B. Graphical visualization of ^31^P NMR measurements for phosphonate **8** **a** (red), phosphate **8** **b** (blue) and phosphoramidate **8** **c** (black).

Next, we aimed to evaluate the charge distribution of peptides **8 a**–**c** at physiological pH. In a previous study, Benkovic and Sampson reported the potentiometrically detected p*K*_a_ values of 9.9 and 2.9 for *N*‐(*n‐*butyl)phosphoramidate for the protonation on nitrogen and one phosphoryl oxygen, respectively,[^17^, ^22^] which gave us an estimate for the p*K*_a_ values of pLys. An elegant technique for the p*K*_a_ determination is NMR titration since this non‐invasive analytical technique enables accurate measurements at desired temperatures at a given pH. Our experimental set up was inspired by the NMR titration conducted by Gamcsik et al. on phosphoramidic acid and other phosphoramides.^[23]^ Samples were prepared at a 1 mm concentration in 40 mm KCl (water + 10 % deuterium oxide) and kept at low temperature to minimize P−N bond hydrolysis for **8 c**. The pH of each sample was adjusted before measurement and checked again afterwards. Proton (^1^H), phosphorous (^31^P) 1D as well as ^1^H‐^31^P‐heteronuclear multiple bond coupling (HMBC) NMR spectra were recorded from pH 2 to pH 11 on a 600 MHz (^1^H frequency) spectrometer at 278 K and subsequently used for calculations of p*K*_a_ values (Figure 2 A, exemplified for **8 a**, see Figure S3 for **8 b**). In order to do so, the chemical shift of ^31^P was plotted against the pH values and resulting curves fitted with sigmoidal functions wherein p*K*_a_ values were obtained from the inflection points (Figure 2 B). We further evaluated the data by plotting and fitting of the ^1^H chemical shifts – ζ‐protons for phosphonate **8 a** and *ϵ*‐protons for phosphate **8 b** and phosphoramidate **8 c** (Figure S4).

**Figure 3:**
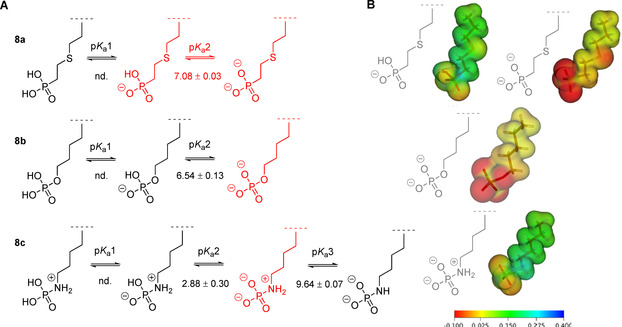
A. Various states of charge and calculated p*K*_a_ values for **8** **a**, **8** **b** and **8** **c**. Highlighted in red is the protonation of each species at physiological conditions. nd.=not determined. B. Calculated ESP maps relying on DFT‐optimized molecular structures of **8** **a**, **8** **b** and **8** **c** at pH 7.0, visualized with Molden 5.9.

Our findings indicated that for each of the three variants the first p*K*_a_ could not be determined, because they were below pH 2. In case of the phosphonate **8 a**, the p*K*_a_ between the mono‐protonated phosphoryl oxygen and the di‐anionic compound was 7.08±0.03 (R^2^=0.9999), thus both species were in a rather balanced equilibrium at physiological conditions (Figure 3). The second p*K*_a_ of phosphate **8 b** at 6.54±0.13 (R^2^=0.9991) pointed towards a more pronounced charge state of −2 at pH 7. For pLys peptide **8 c** it was possible to determine two p*K*_a_ values, one at 2.88±0.30 and the second at 9.64±0.07 (R^2^=0.9995). This result correlates with the complete deprotonation of the phosphoryl oxygens (p*K*_a_2) and the deprotonation of the amine (p*K*_a_3). Comparing the change in ppm (Δppm) at the p*K*_a_ values with those reported by Gamcsik et al. supported our assignments of charge states, whereby a smaller value indicated a protonation change on oxygen, while a larger value correlated with the protonation of nitrogen.^[23]^ Along these lines, we observed Δppm of 5.09 ppm and 8.82 pm for p*K*_a_2 and p*K*_a_3 for **8 c**. This can also be seen in Figure 2 B as the Δppm for **8 a** and **8 b** representing protonation of oxygen are below 3.5 ppm each. Interestingly, increasing pH values resulted in an upfield move of ^31^P chemical shift for phosphonate **8 a** but a downfield move for phosphoramidate **8 c** and phosphate **8 b** (Figure 2 B). This behavior has been described already for adenosine nucleotide analogues in the early 1980s, in which phosphonate derivatives of triphosphates exhibited the same pattern as observed now.^[24]^ The net charges of the side chains of interest of **8 a**, **8 b** and **8 c** at pH 7 are illustrated in Figure 3 A. To visualize the charge distribution, electrostatic potential (ESP) maps were calculated for optimized structures as shown in Figure 3 B (see SI, section 2.10 for details). Even though the relaxed structures had great geometrical similarity, the charge distributions showed clear differences. While the charge density was well spread over the whole molecule with only a slightly negative partial charge at the phosphoryl oxygens in pLys peptide **8 c** as well as the mono‐anion of **8 a**, the di‐anionic species of **8 a** and **8 b** exhibited distinct charge fluctuations across the structures. This comparison indicates that phosphonate **8 a** is the more suitable mimic for pLys at physiological pH.

## Conclusions

In conclusion, we designed both, a phosphonate and a phosphate analogue of pLys, and evaluated their potency to act as pLys mimics by comparing their p*K_a_* values. Both analogues were obtained as suitably protected building blocks, which were used in the straightforward Fmoc‐based SPPS to obtain model peptides for the p*K_a_* measurements. In contrast, a novel Tc‐protected pLys building block failed to efficiently deliver pLys peptides when applied in SPPS. Subsequent stability studies and NMR measurements showed that phosphonate **8 a** is a very promising non‐hydrolyzable mimic of pLys with a comparable charge distribution, thus opening the opportunity of acting as an inhibitor for pLys interacting enzymes. Instead, phosphate **8 b** exhibited a charge distribution at physiological pH, which strongly differs from pLys **8 c**, thereby indicating a lower probability to be recognized by pLys‐selective proteins. Although these experiments have been conducted with model substrates, we believe that these findings point toward very promising pLys analogues with high stability for the generation of pLys‐specific Abs or the screen for binding partners.

## Experimental Section

Detailed experimental procedures, compound characterization and supplementary figures can be found in the supporting information.

## Conflict of interest

The authors declare no conflict of interest.

## Supporting information

As a service to our authors and readers, this journal provides supporting information supplied by the authors. Such materials are peer reviewed and may be re‐organized for online delivery, but are not copy‐edited or typeset. Technical support issues arising from supporting information (other than missing files) should be addressed to the authors.

Supplementary
