# Supplementary material for: Synthesis and Evaluation of Non‐Hydrolyzable Phospho‐Lysine Peptide Mimics
Source: Chemistry. 2020 Dec 7;27(7):2326–31. doi: 10.1002/chem.202003947 (PMC7898648; doi:10.1002/chem.202003947)
Supplement: Supplementary file 1 — Supplementary [file CHEM-27-2326-s001.pdf]

Chemistry–A European Journal

Supporting Information

Synthesis and Evaluation of Non-Hydrolyzable Phospho-Lysine Peptide Mimics

Anett Hauser,[a, b] Eleftheria Poulou,[a] Fabian Müller,[b] Peter Schmieder,[a] and Christian P. R. Hackenberger*[a, b]

Table of Contents

1. **Supplementary Figures** 4
  1.1 **Figure S1** 4
  1.2 **Figure S2** 5
  1.3 **Figure S3** 6
  1.4 **Figure S4** 7
2. **General Information** 8
  2.1 **Reagents and Solvents** 8
  2.2 **Peptide Synthesis** 8
  2.3 **Analytical UPLC** 9
  2.4 **TLC analysis** 10
  2.5 **Purification** 10
  2.6 **NMR** 10
  2.7 **Photodeprotection** 11
  2.8 **Phosphatase Activity Assay** 12
  2.9 **UV/Vis Spectroscopy** 12
  2.10 **Electrostatic Potential Maps** 13
3. **Organic Synthesis** 14
  3.1 **Synthesis of homocysteine-derived phosphonate 1** 14
    3.1.1 **Synthesis overview** 14
    3.1.3 **Dibenzyl vinylphosphonate (4)** 15
    3.1.4 **(((9*H*-fluoren-9-yl)methoxy)carbonyl)-*L*-homocysteine (5)** 15
    3.1.5 ***N*-(((9*H*-fluoren-9-yl)methoxy)carbonyl)-*S*-(2-(bis(benzyloxy)phosphoryl)ethyl)-*L*-homocysteine, Fmoc-hCys(EtPO(OBn)$_2$)-OH (1)** 16
  3.2 **Synthesis of norleucine-derived phosphate 2** 17
    3.2.1 **Synthesis overview** 17
  3.3 **Peptide synthesis with building blocks 1 and 2** 18
    3.3.1 **Synthesis of ^{Ac}Tyr-hCys(EtPO(OH)$_2$)-Gly^{CONH2} (8a)** 18
    3.3.2 **Synthesis of ^{Ac}Tyr-Nle(OPO(OH)$_2$)-Gly^{CONH2} (8b)** 19
  3.4 **Fmoc-based synthesis of caged phospho-lysine peptide** 20
    3.4.1 **Synthesis of the Tc-protected building block 3** 20
    3.4.2 **Peptide synthesis with building block 3 (peptide 12)** 24
  3.5 **Synthesis of phospho-lysine peptide 8c via the Staudinger-phosphite reaction** 25
    3.5.1 **Synthesis overview** 25
    3.5.2 **1-(2-nitrophenyl)ethanol (14)** 25

3.5.3 Tris(1-(2-nitrophenyl)ethyl) phosphite (15) 26
3.5.4 ^{Ac}Tyr-Lys(N_3)-Gly^{CONH2} (16) 27
3.5.5 ^{Ac}Tyr-Lys(NPO(ONPE)$_2$)-Gly^{CONH2} (13) 27
3.5.6 ^{Ac}Tyr-pLys-Gly^{CONH2} (8c) 28
4. UPLC/UV Chromatograms at 220 nm 29
5. NMR Spectra 32
6.References 48

1. Supplementary Figures

1.1 Figure S1

Side products occurred during SPPS using building block **3** and corresponding yields. While the desired peptide **12** was obtained, a considerable P–N bond hydrolysis was observed, as well as another side product, in which the detected mass would correspond to a Tyr addition and subsequent acetylation at the lysine side-chain.

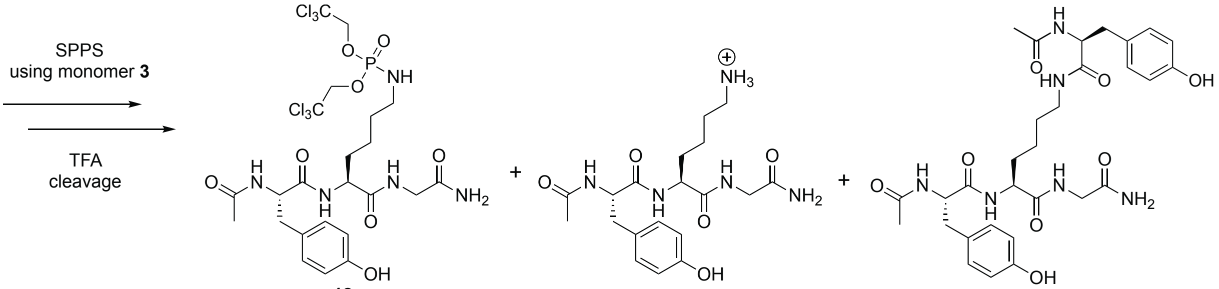

12 14% | **P−N hydrolysis** 11% | **Tyr addition** 7%

12 : P−N hydrolysis : Tyr addition = 1.3 : 1 : 0.5

1.2 Figure S2

Treatment of peptides **8a-c** with alkaline phosphatase (ALP). Figure S2 shows the amount of enzymatically hydrolyzed substrate, calculated by subtraction of detected inorganic phosphate (P_i) in blank reactions (without enzyme) from the reactions with enzyme added. Herein, we observed dephosphorylation for both peptides **8b** and **8c**, which indicated susceptibility of the phosphate analogue towards phosphatases. As expected, no P_i was released from phosphonate **8a** during incubation with ALP.

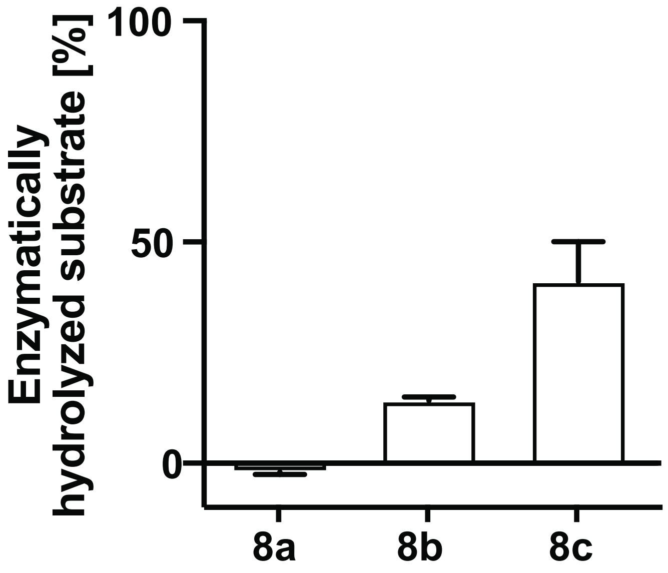

1.3 Figure S3

NMR titration of peptide **8b**. Herein, the signals extracted from the ^{1}H-^{31}P-HMBC experiments in distinct pH values are shown. Conditions: 1 mM peptide in 40 mM KCl in H2O + 10%D2O was measured at 278 K in a 600MHz (^{1}H frequency) spectrometer at pH values from 2 to 11.
A. Overlay of ^{31}P NMR 1D spectra at different pH values.
B. Extracted correlation signals between ε-protons and phosphorous of **8b** from ^{1}H,^{31}P-HMBC NMR experiments.

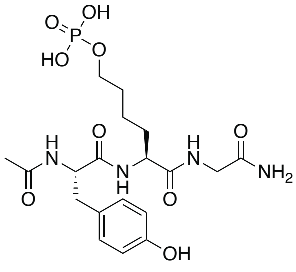

8b

A

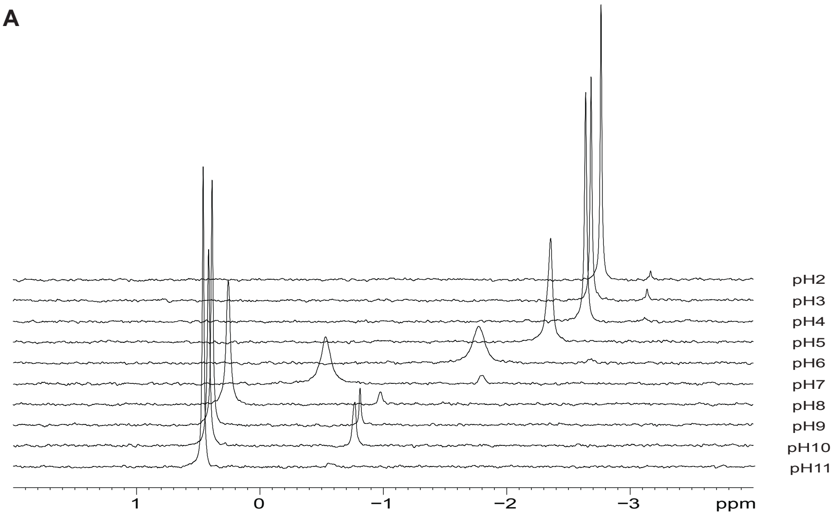

B

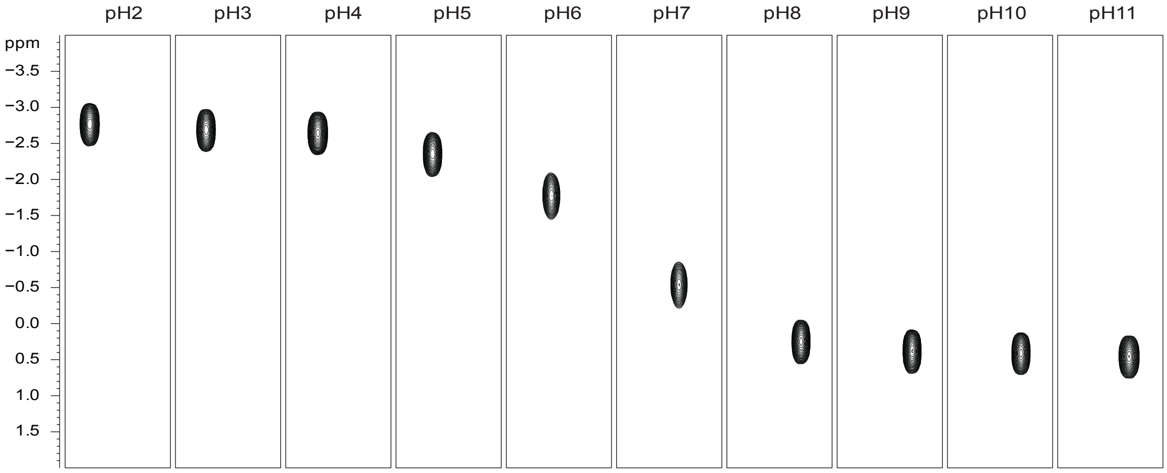

1.4 Figure S4

Graphical visualizations of NMR measurements of ^{1}H chemical shifts of phosphonate **8a** (ζ-protons), phosphate **8b** (ε-protons) and phosphoramidate **8c** (ε-protons).

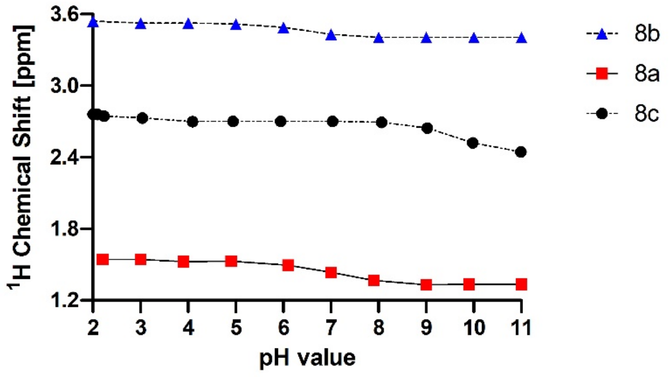

2. General Information

2.1 Reagents and Solvents

Reagents and solvents were, unless stated otherwise, commercially available as reagent grade and did not require further purification. Chemicals were purchased either from Sigma-Aldrich Chemie GmbH (Munich, Germany) or TCI (TCI Deutschland GmbH, Eschborn, Germany). Resins, coupling reagents and amino acids suitable for SPPS were purchased either from IRIS Biotech GmbH (Marktrewitz, Germany) or Novabiochem© (Merck KGaA, Darmstadt, Germany). Aliquots from *bovine alkaline phosphatase* (ALP, Sigma-Aldrich) were kindly provided by Prof. Fiedler. All water- and air-sensitive reactions were performed under Schlenk conditions.

2.2 Peptide Synthesis

Peptides were prepared using the Fmoc solid phase strategy by manual peptide synthesis in 8 mL Extract CleanTM reservoirs (Grace Davison Discovery Sciences, Deerfield, Illinois, USA) of 40 mL reactors (Activotec, Cambridge, UK) both equipped with fitting teflon frits.

Coupling of first amino acid on Rink amide resin

Swelling: 3.5 g Rink Amide resin (Novabiochem, 100-200 mesh, Fmoc on, average loading: 0.73 mmol x g^{-1}) were swollen in DMF for 45 min.
Fmoc deprotection: After 2x 10 min shaking, each with 10 mL of piperidine (Pip): DMF (1:4, v/v) the resin was washed with 5x DMF, 5x DCM, 5x DMF.
Coupling: A 0.1 M solution of 0.4 eq. (1 mmol) Fmoc-protected Glycine, 0.4 eq (1 mmol) PyBop and 0.8 eq. (2 mmol) DIPEA in DMF was added to the resin. After 2 h of shaking at r.t., the resin was washed (5x DMF, 5x DCM, 5x DMF).
Capping: A solution of Ac2O:2,6-lutidine: DMF (5:6:89, v/v/v, 10 mL) was added and the mixture shaken for 10 min. The resin was washed (5x DMF, 10x DCM) and dried completely under reduced pressure. The loading was determined photometrically as described in the UV/Vis spectroscopy section. For every peptide synthesis described herein, an Fmoc-Gly pre-loaded resin **17** was prepared with a determined loading of 0.4385 µmol^{x}mg $^{-1}$

Manual peptide synthesis

Swelling: The corresponding amount of resin for a 100 µmol scale was swollen in DMF (2000 µL) for 30min.

Fmoc deprotection: Pip:DMF (1:4, v/v, 1000 µL) was added to the resin. After 5 min, the solution was discarded and another portion of Pip:DMF (1:4, v/v, 1000 µL) was added to the resin. After 5 min, the solution was discarded and the resin washed with DMF, DCM, DMF (3x 1000 µL each).

Coupling: Aas were dissolved together with HCTU (500 µmol, 5 eq.) and Oxyma (500 µmol, 5 eq.) to a 0.2M solution in DMF. Directly before adding the mixture to the resin, DIPEA (1000 µmol, 10 eq.) was added. The resulting reaction mixture was shaken for 45 min at r.t., the resin then filtered and washed with DMF, DCM, DMF (3x 1000 µL each). Fmoc-Lys(N3)-OH, Fmoc-hCys(PO(OBn)$_2$)-OH **1**, Fmoc-Nle(OPO(OH)(OBn))-OH **2**, Fmoc-Lys(PO(OTc)$_2$)-OH **3** were incorporated by using 2 eq. AA, 1.95 eq. HATU and 4 eq. DIPEA in DMF and reacting the mixture for 2 h at r.t.

Acetylation: *N*-terminal acetylation was performed by treating the resin with a mixture of Ac2O:2,6-lutidine: DMF (5:6:89, 1 mL) for 10 min at r.t., after which the resin was washed with DMF, DCM, DMF (3x 1000 µL each).

Final cleavage: The resin was either washed with DCM (10x 1000 µL) or used dry and treated with 4 mL of the cleavage cocktail (TFA: TIS: H2O – 95:2.5:2.5, v/v/v) for 2 h.

Peptides containing the hCys-derivative **1** were treated for 105 min with the standard cleavage cocktail, before a solution of 60 µL EDT and 300 µL TMSBr was added and the mixture shaken for further 15 min. The resin was filtered off, the TFA filtrate collected in a 10-fold excess of deep-frozen Et2O and let sit for precipitation in the freezer. After at least 15 min, the mixture was centrifuged, the solution decanted, the precipitate dried under nitrogen and re-dissolved in ACN/H_2O for UPLC analysis and preparative HPLC.

2.3 Analytical UPLC

UPLC®-UV traces for peptides and small molecules were obtained on an ACQUITY H-class instrument (Waters Corporation, Milford, Massachusetts, USA) equipped with an ACQUITY UPLC®-BEH C18 1.7 &m, 2.1x50 mm column (Waters Corporation), applying a flow rate of 0.6 ml x min^{-1} and using eluents A (99.9% H_2O, 0.1% TFA) and B (99.9% ACN, 0.1% TFA) in the corresponding linear gradient. UPLC-UV chromatograms were recorded at 220 nm.

Gradients: I 5% to 95% B in 13 min

II 0.5% to 60% B in 13 min

High resolution masses were recorded on an ACQUITY H-class instrument (Waters Corporation) equipped with an ESI-MS Xevo® G2-XS QTof spectrometer (Waters Corporation).

2.4 TLC analysis

The thin layer chromatography (TLC) was performed on silica gel plates with fluorescence indicator F254 (Merck KGaA). Detection was performed at 254 or 366 nm. Compounds without any chromophore were stained with any of staining solutions such as potassium permanganate solution, ninhydrin or vanillin reagent.

2.5 Purification

Peptidic substrates were purified by preparative, semi-preparative or analytical HPLC, performed either on a Gilson PLC 2020 system (Gilson Inc., Middleton, Wisconsin, USA), a Shimadzu Prominence 20A system or a Shimadzu Prominence 8A system (both Shimadzu Corporation, Kyoto,Japan) equipped with columns as followed: preparative column – Nucleodur C18 HTec, 5 µm, 250x32 mm; semi-preparative column –Nucleodur C18 HTec, 5 µm, 250x21 mm; analytical column– Nucleodur C18 HTec, 5 µm, 250x10 mm (all columns purchased from Macherey-Nagel, GmbH & Co. KG, Düren, Germany). Eluents A (99.9%H_2O, 0.1% TFA) and B (B**I**: 99.9% ACN, 0.1%TFA or B**II**:9.9% ACN, 20% H_2O, 0.1% TFA) were applied in the corresponding linear gradient. Peak detection was performed at 220 nm. Small molecules were purified by silica gel column chromatography (VWR Chemicals, Normasil 60 Å, 40–63 µm). The samples were applied pre-absorbed on silica gel or, if liquid, directly diluted with suitable solvents.

2.6 NMR

NMR spectra were recorded either with a Bruker AV III HD 300MHz spectrometer or a Bruker AV III600MHz spectrometer (both Bruker Corporation, Billerica,Massachusetts USA) at ambient temperature if not stated differently. The chemical shifts for proton signals (^{1}H) are reported in ppm relative to the shift of tetramethylsilane. The chemical shifts for phosphorous signals (^{31}P) are reported relative to the signal of phosphoric acid (H_3PO_4) using indirect calibration.

NMR titration experiments

Typically, samples were dissolved in 40 mM KCl to a concentration of 1 mM. The solutions were immediately cooled in an ice-water bath. The pH meter was calibrated at 20 °C; however, all sample readings were performed in the ice-water bath. The pH of the samples was measured before and after the NMR experiment and agreed to within 0.01 pH unit. The pH values used for calculations are the average of the two readings and are not corrected for the temperature. Solutions were then transferred into a 5 mm sample tubes and sealed capillaries filled with D_2O were added. The samples were measured sequentially using an automatic sample changer and were thus kept a room temperature for most of the time, the temperature for the experiments was adjusted only after insertion into the magnet.

Experiments were performed at 278 K on a AV-III-600 NMR-spectrometer (600 MHz ^1H frequency, Bruker Biospin, Karlsruhe, Germany) equipped with a 60-slot BACS sample changer using a QCI cryoprobe equipped with a cooled ^{31}P coil and preamp and a one-axis self-shielded gradients. The samples were inserted sequentially and 10 minutes were given for temperature equilibration. Temperatures had been calibrated using d_4-methanol. [1] Topspin 3.2 was used to control the spectrometer. One-dimensional (1D) ^{31}P experiments were recorded using 64k complex points with an acquisition time $t_{P,max}$ = 2.62 sec (i.e. a spectral window of 25,000 Hz was used), a relaxation delay of 2.5 sec and 256 scans. Two-dimensional (2D) ^1H,^{31}P-HMBC experiments[2, 3] were performed using a gradient version of the experiment in which a WATERGATE[4] water suppression had been implemented. 2,048 (^1H) · 64 (^{31}P) complex points were acquired, with acquisition times $t_{H,max}$ = 204.8 ms and $t_{P,max}$ = 6.4 ms (i.e. a spectral window of 10,000 Hz was used in each dimension) and 16 scans. NMR data were processed and spectra viewed using topspin 3.2 (Bruker Biospin).

2.7 Photodeprotection

UV-irradiation was carried out with a Hg (Xe) arc lamp (LOT-Quantum Design GmbH, Darmstadt, Germany) using a 297 nm filter with 15% transmission (Andover Inc., Salem, New Haven,USA). Samples were dissolved in MeOH at a concentration of 7.5 mM, positioned in 20 cm distance to the source and irradiated while stirring. The deprotection progress was followed by UPLC analysis. Upon complete conversion, a 10-fold excess of deep-frozen Et_2O was added and the mixture let sit in the freezer for 10 min. After centrifugation, the liquid phase was discarded and the precipitate re-suspended in the same amount of deep-frozen Et_2O. The mixture was kept in the freezer again for 10 min, centrifuged, decanted and the precipitate dried under reduced pressure for 15 min. Deprotected pLys substrates were stored in the freezer until applied in the assay.

2.8 Phosphatase Activity Assay

Phosphatase activities were determined on a SAFIRE^2 microplate reader (TecanGroup Ltd., Männedorf, Switzerland) by photometric detection at 360 nm of released inorganic phosphate with the EnzCheck™ Phosphatase Assay Kit (ThermoFisher Scientific™, Waltham, Massachusetts, USA) following the protocol for enzymatic kinetics.[5] Briefly, substrates were incubated at a concentration of 100 µM in 50 mM Tris-HCl buffer containing 1 mM $MgCl_2$ and 1 mM $ZnCl_2$ at pH 7.8 in the presence or absence of 0.05 U ALP for the overall phosphate release (enzymatic plus background reaction, $[E{+}BG]_{wBL}$) or the non-enzymatic hydrolysis ($[BG]_{wBL}$), respectively.

The absorbance values of microplate, buffer, EnzCheck™ reagent and ALP were determined in separated wells without adding substrate to the solution, considered as baseline ($[BL]_{w/ALP}$ and $[BL]_{w/oALP}$). Reactions were run for 90 min in total, UV-absorbance was measured every 10 min. The enzymatic hydrolysis yield $[E]$ was determined by substraction of $[BG]_{w/oBL}$ from $[E{+}BG]$w/oBL.

$$[E\,] = [E + BG]_{w/oBL} - [BG]_{w/oBL}$$

$$= ([E + BG]_{w/BL} - [BL]_{w/ALP}) - ([BG]_{w/BL} - [BL]_{w/oALP})$$

2.9 UV/Vis Spectroscopy

UV/Vis spectra and absorbance values were determined either on a V-630 spectrophotometer at r.t. or a V-550 UV/Vis spectrophotometer equipped with an ETC-505T temperature controller at 20 °C (both Jasco, Tokyo, Japan).

Determination of resin loading for peptide synthesis

1-2 mg dried resin were weighed into a microcentrifuge tube covered with 1 mL Fmoc deprotection solution (Pip:DMF, 1:4, v/v). After 10 min of shaking at r.t., the loading was determined in a 1 mL quartz cuvette. An aliquot of the deprotection solution was diluted to such an extent that the expected resulting absorbance was approx. 0.3. From the measured absorbance the loading was calculated by the help of the following equations:

$$A_\lambda = \varepsilon_\lambda \cdot c \cdot d$$

A: Absorbance at given wavelength, ε: molar attenuation coefficient at given wavelength in $L \cdot mol^{-1} \cdot cm^{-1}$ (ε_{301nm} = 7800 $L \cdot mol^{-1} \cdot cm^{-1}$ for 1-((9H-fluoren-9-yl)methyl)piperidine), c: concentration in $mol \cdot L^{-1}$, d: cuvette length in cm.

$$Lresin = \frac{A \times Vdeprotection \times Vcuvette}{\varepsilon_{301nm} \times d \times Valiquot \times mresin}$$

$Lresin$: loading in $\mu mol \cdot mg^{-1}$; *A*: Absorbance at 301 nm; $V_{deprotection}$: volume of Fmoc deprotection solution in µL (1000 µL); $V_{cuvette}$: total volume in cuvette for measuring in µL; ε_{301nm} = 7800 $\mu L \cdot \mu mol^{-1} \cdot cm^{-1}$; d: cuvette length in cm; m_{resin} : weighed amount in mg.

2.10 Electrostatic Potential Maps

Electrostatic potential (ESP) maps were calculated relying on density functional theory (DFT) optimized molecular structures. The exchange correlation functional B3LYP[6-8] together with the polarized triple zeta basis set def2-TZVPP[9, 10] on all atoms was employed and subsequent harmonic vibrational frequency analysis was carried out in order to check if a real local minimum on the potential energy surface was found. From the created electron densities, the electrostatic potential energy (in a.u.) was calculated and mapped on the respective density plot for a contour value of 0.01. The Turbomole program package V7.0.2[11-13] was used for all DFT electronic structure and geometry optimizations. ESP evaluations and visualizations were done with Molden 5.9.[14, 15]

3. Organic Synthesis

3.1 Synthesis of homocysteine-derived phosphonate 1

3.1.1 Synthesis overview

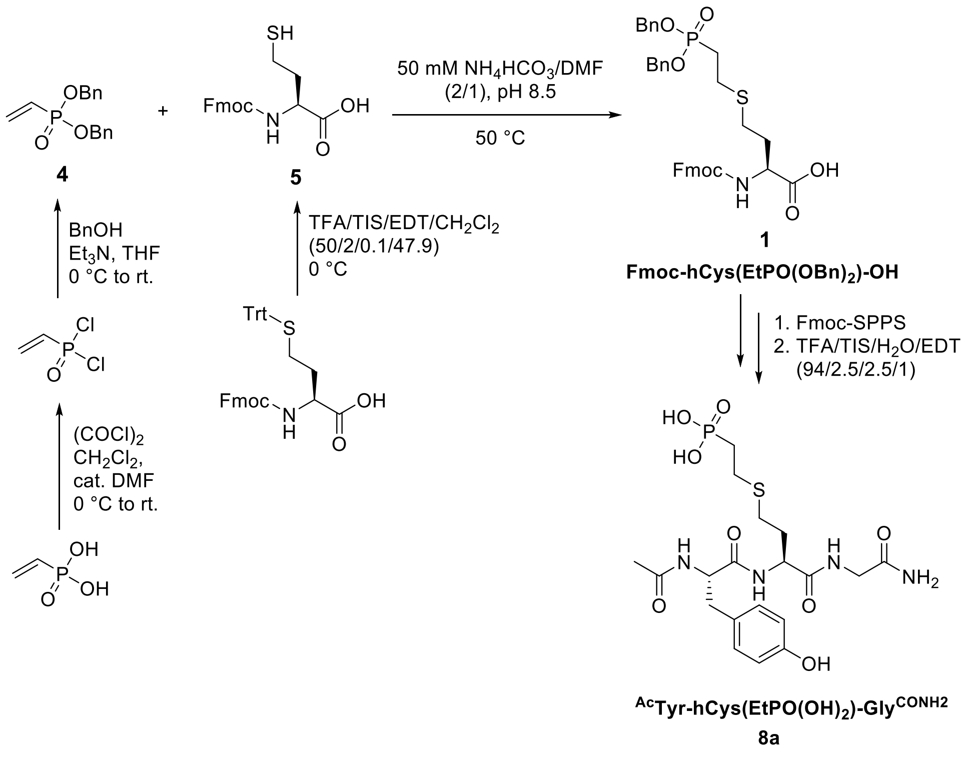

3.1.2 Vinylphosphonic dichloride (crude)

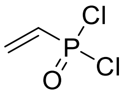

Vinylphosphonic dichloride was synthesized as described before with minor adjustments. Briefly, in a heated Schlenk flask under Ar, 19 mL of dry DCM were cooled in an ice bath for 5 min.1.34 mL (17.3 mmol) vinylphosphonic acid were added and cooled for 5 min before 5 drops of dry DMF were added. Under vigorous stirring, 4.47 mL (52.1 mmol, 3 eq.) oxalyl dichloride were added dropwise and the resulting orange mixture kept in the ice bath for further 15 min and then at rt. ovn. After 17.5 h solvents and oxalyl dichloride were evaporated under reduced pressure. The crude product was obtained as an orange-brown oil and used for the next step without further purification. ^{1}H NMR (300 MHz, $CDCl_3$) δ 6.65 –6.61 (m, 1H), 6.52–6.21 (m, 2H). ^{31}P NMR (122 MHz, $CDCl_3$) δ 31.31.

3.1.3 Dibenzyl vinylphosphonate (4)

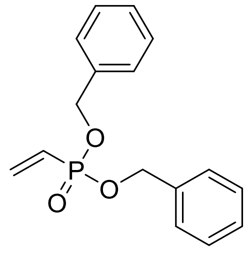

In a heated flask and in Ar atmosphere, 50 mL dry THF were pre-cooled. Subsequently, 4.5 mL (43.25 mmol, 2.5 eq.) benzyl alcohol and 7.2 mL (52.1 mmol, 3 eq.) Et_3N were added and cooled as well. 17.3 mmol (1 eq.) vinylphosphonic dichloride were dissolved in 10mL dry THF and added dropwise to the pre-cooled mixture, upon which a white precipitate was formed. The reaction was stirred for 15 min in ice bath and ovn at r.t. Next day, the precipitate was filtered off, solvents evaporated under reduced pressure and the crude product purified by silica column chromatography (100% DCM → DCM/EE 8/2, product eluting at 10% EE). The product was obtained as orange oil (3.24 g, 11.4 mmol, 66%). ^1H NMR (600 MHz, CD_3CN) δ 7.43 – 7.31 (m, 10H), 6.29 – 6.05 (m, 3H), 5.07 – 4.96 (m, 4H). ^{31}P NMR (243 MHz, CD_3CN) δ 14.70, 14.67. ^{13}C NMR (151 MHz, CD_3CN) δ 139.44, 139.30, 138.27, 138.22, 137.19, 137.13, 131.83, 131.79, 131.58, 131.53, 131.18, 131.15, 131.12,130.76, 130.73, 130.56, 130.54, 130.51, 130.48, 130.45, 130.12, 130.09, 130.06, 129.79, 128.69, 128.58,127.50, 70.76, 69.80, 69.77, 68.79. R*f* (DCM/EE 7/3, $KMnO_4$ stain) = 0.50. HR-MS for $C_{16}H_{17}O_3P$: *m*/*z* calc. $[M+H^+]^+$ = 289.0988, *m*/*z* obs. $[M+H^+]^+$ = 289.0994.

3.1.4 (((9*H*-fluoren-9-yl)methoxy)carbonyl)-*L*-homocysteine (5)

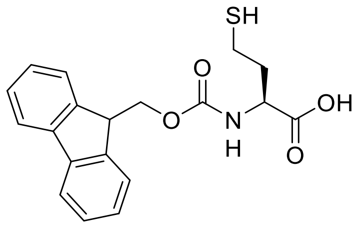

At 0 °C, 480 mg (0.8 mmol) Fmoc-hCys(Trt)-OH were dissolved in 2.4 mL DCM and 2.6 mL of a mixture of TFA: TIS: EDT (96:3.8:0.2, v/v/v) added. Within 1 min the mixture turned yellow and back to colourless. UPLC analysis after 7 min indicated full conversion. Solvents were evaporated with nitrogen flush, while keeping the mixture in ice bath. Immediate purification on preparative HPLC gave 240 mg (0.67 mmol, 84%) of desired compound as white powder. ^1H NMR (600 MHz, CD_3CN) δ 7.92 (d, *J* = 7.5 Hz, 2H), 7.76 (t, *J* = 7.0 Hz, 2H), 7.51 (t, *J* = 7.5

Hz, 2H), 7.43 (tt, J = 7.5, 1.5 Hz, 2H), 6.07 (d, J = 8.4 Hz, 1H), 4.44 (d, J = 7.2 Hz, 2H), 4.39 (td, J = 8.8, 4.5 Hz, 1H), 4.33 (t, J =7.0 Hz, 1H), 2.71 – 2.57 (m, 2H), 2.16 – 2.03 (m, 2H). ^{13}C NMR (151MHz, CD_3CN) δ 175.35, 158.97,146.81, 143.89, 131.00, 130.95, 130.40, 130.35, 129.94, 129.89, 129.33, 129.29, 128.46, 127.38, 123.27, 123.22, 122.22, 122.17, 69.08, 55.53, 54.59, 50.22, 49.36, 39.08, 38.21, 37.36, 24.21, 23.25. HR-MS for $C_{19}H_{19}NO_4S$: *m/z* calc. $[M+H^+]^+$ = 358.1108, *m/z* obs. $[M+H^+]^+$ = 358.1119.

3.1.5 *N*-(((9*H*-fluoren-9-yl)methoxy)carbonyl)-*S*-(2-(bis(benzyloxy)phosphoryl)ethyl)-*L*-homocysteine, Fmoc-hCys(EtPO(OBn)$_2$)-OH (1)

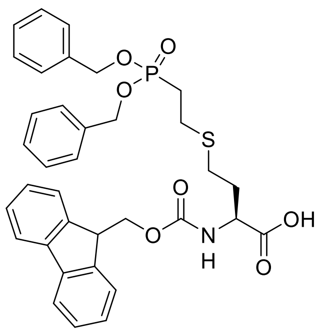

Thiol addition of Fmoc-hCys-OH **5** with alkene **4** was performed at basic pH as followed. 100 mg (280 µmol) AA and 242 mg (840 µmol, 3 eq.) alkene were dissolved in 1.5 mL DMF. After addition of 5 mL 50 mM AmBic buffer, pH 8.5 and pH adjustment to 8.7, the formed precipitate was redissolved with 1.2 mL additional DMF (final concentration of AA 45 mM). The reaction was stirred at 45 °C for 6 h until no starting material could be detected by UPLC analysis anymore. Solvents were evaporated under reduced pressure and by lyophilization and the crude mixture purified by preparative HPLC. 122.9 mg (190.4 µmol, 68%) of desired product were obtained as white powder. ^{1}H NMR (600 MHz, CD_3CN) δ 7.81 (d, J = 7.5 Hz, 2H), 7.65 (dd, J = 7.5, 4.5 Hz, 2H), 7.44 – 7.26 (m, 14H), 6.17 (d, J = 8.4 Hz, 1H), 5.10 – 4.92 (m, 4H), 4.31 (d, J = 6.9 Hz, 2H), 4.29 - 4.24 (m, 1H), 4.20 (t, J = 7.0 Hz, 1H), 2.71 – 2.58 (m, 2H), 2.59 - 2.50 (m, 2H), 2.19 – 1.83 (m, 4H). ^{31}P NMR (243 MHz, CD_3CN) δ 30.15. ^{13}C NMR (151 MHz, CD_3CN) δ 173.07, 156.28, 144.11, 144.01, 141.13, 136.53, 136.45, 128.61, 128.44, 127.99, 127.73, 127.14, 125.24, 120.00, 67.46, 67.44, 67.37, 67.35, 66.35, 52.79, 47.02, 31.04, 27.44, 27.18, 25.40, 23.88, 23.83. HR-MS for $C_{35}H_{36}NO_7PS$: *m/z* calc. $[M+H^+]^+$ = 646.2043, *m/z* obs. $[M+H^+]^+$ = 646.2042.

3.2 Synthesis of norleucine-derived phosphate 2

3.2.1 Synthesis overview

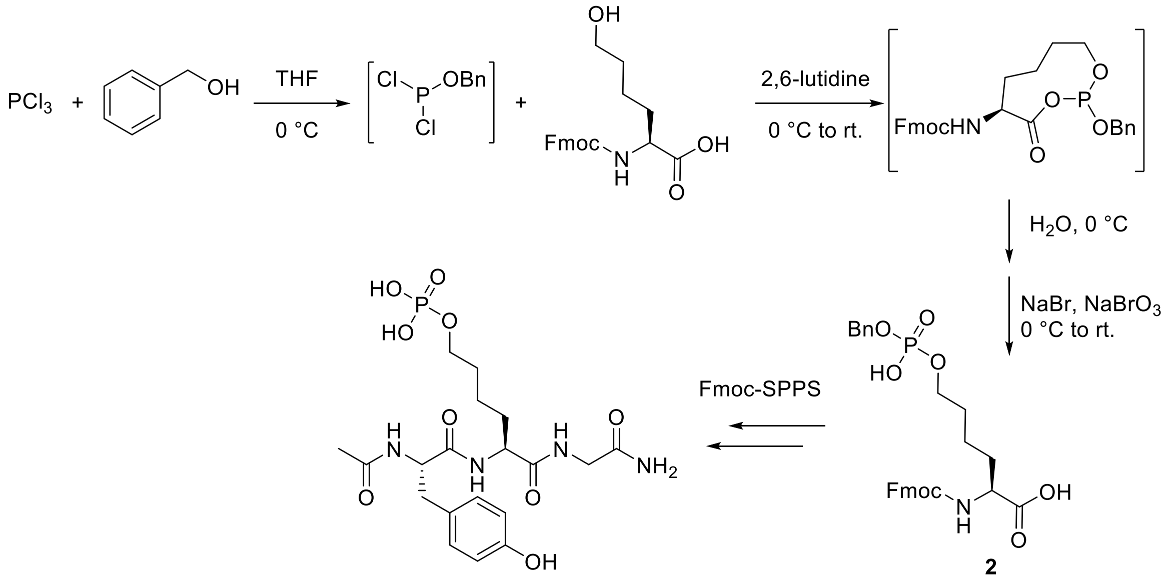

2
Fmoc-Nle(OPO(OH)(OBn))-OH

AcTyr-Nle(OPO(OH)$_2$)-Gly^{CONH2}
8b

3.2.2 (2*S*)-2-((((9*H*-fluoren-9-yl)methoxy)carbonyl)amino)-6-(((benzyloxy)(hydroxy)phosphoryl)oxy)hexanoic acid, Fmoc-Nle(OPO(OH)(OBn))-OH (2)

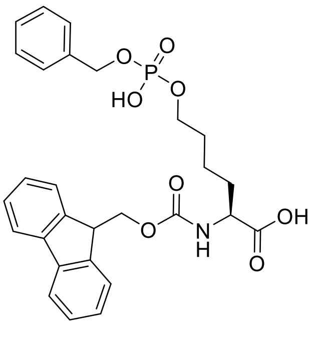

Fmoc-Nle(OPO(OH)(OBn))-OH **2** was prepared as previously described for the pSer derivative.[16] Briefly, in a heated flask and under Ar, 16.5 mL dry THF were cooled in an ice bath for 5 min. 201 µL (2.29 mmol, 1.7 eq) phosphorous trichloride were added and cooled for 2 min as well. While cooling, 238 µL (2.29 mmol, 1.7 eq.) benzyl alcohol were added dropwise and the resulting solution kept at 0 °C for 5 min. Since ^{31}P NMR indicated some PCl_3 left, further 0.3 eq. (0.4 mmol, 42 µL) benzyl alcohol were added and the mixture stirred for 5 min at 0 °C. Upon the addition of 490 µL (4.23mmol, 3.1 eq.) 2,6-lutidine, a white precipitate was formed, to which a mixture of 500 mg (1.35 mmol, 1 eq.) Fmoc-Nle(6-OH)-OH, 2.8 mL dry THF and 167 µL (1.44 mmol, 1.07 eq.) 2,6-lutidine was dropped within 5min. The flask was washed

with 0.6 mL dry THF to give a final concentration of AA of 65 mM. The reaction was kept for 10 min cooled and then stirred at r.t. until UPLC analysis indicated full consumption of the AA (90 min). Successively, 1.7 mL H2O, 321 mg (3.12 mmol, 2.3 eq) sodium bromide and 510 µL of a 20% sodium bromate solution (containing 102 mg, 0.7 mmol, 0.51 eq $NaBrO_3$) were added, upon which the mixture turned orange. After 5 min cooling with ice, the reaction was stirred at r.t. for 4 h, when UPLC analysis indicated formation of the desired product. Remaining oxidation reagent was quenched with 10 mL of a $Na_2S_2O_5$ solution as could be observed by destaining of the mixture. The crude product was obtained after evaporation of the solvents under reduced pressure and with lyophilization. 561 mg (1.04 mmol, 77%) of monobenzyl/Fmoc protected phospho-AA **2** were obtained after purification using preparative HPLC. ^{1}H NMR (600 MHz, CD_3CN) δ 7.81 (d, *J* = 7.6 Hz, 2H), 7.65 (t, *J* = 6.7 Hz, 2H), 7.41 – 7.37 (m,2H), 7.37 – 7.34 (m, 5H), 7.33 – 7.29 (m, 2H), 6.05 (s, 1H), 5.02 (dd, *J* = 8.1, 2.2 Hz, 2H), 4.33 – 4.28 (m, 2H), 4.20 (t, *J* = 7.0 Hz, 1H), 4.12 (d, *J* = 7.5 Hz, 1H), 3.98 (q, *J* = 6.6 Hz, 2H), 1.84 – 1.55 (m, 4H), 1.41 (dq, *J* = 15.7, 7.4 Hz, 2H). ^{31}P NMR (243 MHz, CD_3CN) *δ* 3.40. ^{13}C NMR (151 MHz, CD_3CN) δ 176.05, 160.46, 160.19, 159.00, 146.78, 143.85, 138.96, 131.84, 131.79, 131.69, 131.13, 130.99, 130.94, 130.77, 130.73, 130.62, 130.39, 130.35, 130.06, 129.93, 129.88, 129.33, 129.29, 128.45, 127.40, 123.26, 123.20, 122.20, 122.15, 71.61, 70.62, 70.06, 69.10, 56.90, 55.98, 50.17, 49.30, 32.03, 24.21. HR-MS for $C_{28}H_{30}NO_8P$: *m/z* calc. $[M+H^+]^+$ = 540.1782, *m/z* obs. $[M+H^+]^+$ = 540.1784.

3.3 Peptide synthesis with building blocks 1 and *2*

3.3.1 Synthesis of ^{Ac}Tyr-hCys(EtPO(OH)$_2$)-Gly^{CONH2} (8a)

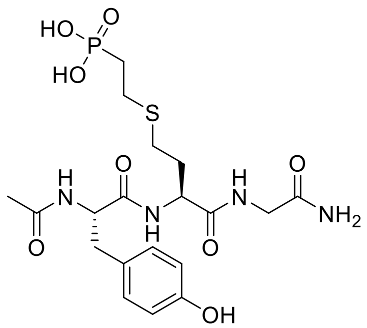

Peptide **8a** was synthesized as described in section 2.2 on a 50 µmol scale with resin **17**. Incorporation of building block **1** was conducted as followed: 2 eq. AA (100 µmol, 65 mg) and 1.95 eq. HATU (97.5 µmol, 37.1 mg) were dissolved in 500 µL DMF to give a 0.2 M solution. Directly before adding the mixture to the Fmoc-deprotected resin, 34 µL (4 eq., 200 µmol) DIPEA were added. The mixture was shaken at r.t. for 2 h, when the TNBS test indicated full conversion. The peptide was capped before continuation of the peptide coupling. The crude product was purified on an analytical HPLC column to give 11.7 mg (23.5 µmol, 47% with regard to initial resin loading) white powder of desired peptide **8a**. ^{1}H NMR (600 MHz, H_2O+10% D_2O, pH 2) δ 8.27 (d, *J* = 7.5 Hz, 1H), 8.13 (d, *J* = 5.8 Hz, 1H), 7.31 (t, *J* = 6.2 Hz, 1H), 7.18 (s, 1H), 6.89 (s, 1H), 6.83 (d, *J* = 8.5 Hz, 2H), 6.51 (d, *J* = 8.5 Hz, 2H), 4.10 (m, 1H), 3.58 – 3.39 (m, 2H), 2.65 (ddd, *J* = 70.4, 13.8, 7.9 Hz, 2H), 2.40 – 2.32 (m, 2H), 2.14 (ddt, *J* = 96.9, 14.6, 7.8 Hz, 2H), 1.85 (s, 1H), 1.66 (s, 3H), 1.64 (s, 1H), 1.57 (dtt, *J* = 18.4, 8.9, 3.8 Hz, 3H). ^{31}P NMR (243 MHz, H_2O+10% D_2O, pH 2) δ 23.13. ^{13}C NMR (600 MHz, D_2O) δ 176.90, 176.54, 176.50, 176.04, 157.29, 133.31, 130.46, 118.24, 118.19, 58.37, 55.18, 44.83, 38.69, 32.70, 30.83, 29.96, 29.67, 27.27, 27.25, 24.26. HR-MS for $C_{19}H_{29}N_4O_8PS$: *m/z* calc. $[M+H^+]^+$ = 505.1517, *m/z* obs. $[M+H^+]^+$ = 505.1521. t_R (gradient **II**) = 3.691 min.

3.3.2 Synthesis of ^{Ac}Tyr-Nle(OPO(OH)$_2$)-Gly^{CONH2} (8b)

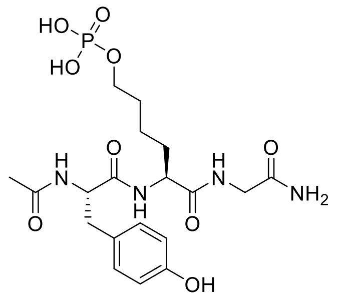

Peptide **8b** was synthesized as described in section 2.2 on a 50 µmol scale with resin **17**. Incorporation of building block **2** was conducted as followed: 2 eq. AA (100 µmol, 54 mg) and 1.95 eq. HATU (97.5 µmol, 37.1 mg) were dissolved in 500 µL DMF to give a 0.2 M solution. Directly before adding the mixture to the Fmoc-deprotected resin, 34 µL (4 eq., 200 µmol) DIPEA were added. The mixture was shaken at r.t. for 2 h, when the TNBS test indicated full conversion. The peptide was capped before continuation of the peptide coupling. The crude product was purified on an analytical HPLC column to give 19.3 mg (39.5 µmol, 79% with regard to initial resin loading) white powder of desired peptide **8b**. ^{1}H NMR (600 MHz, D_2O) δ 7.13 – 7.07 (m, 2H),

6.82 – 6.77 (m, 2H), 4.51 – 4.45 (m, 1H), 4.21 – 4.16 (m, 1H), 3.88 – 3.83 (m, 2H), 3.78 (dq, J = 24.3, 1.7 Hz, 2H), 2.97 – 2.89 (m, 2H), 1.93 (s, 3H), 1.80 – 1.52 (m, 4H), 1.37 – 1.22 (m, 2H). ^{31}P NMR (243MHz, D_2O) δ 0.28. ^{13}C NMR (600 MHz, D2O) δ 176.87, 176.75, 176.63, 176.39, 157.24, 133.29, 130.57, 118.19, 68.94, 68.90, 58.14, 56.46, 44.81, 38.80, 32.83, 31.82, 31.78, 24.26, 23.91. HR-MS for $C_{19}H_{29}N_4O_9P$: m/z calc. $[M+H^+]^+$ = 489.1745, m/z obs. $[M+H^+]^+$ = 489.1740. t_R (gradient **II**) = 1.591 min.

3.4 Fmoc-based synthesis of caged phospho-lysine peptide

3.4.1 Synthesis of the Tc-protected building block 3

3.4.1.1 Synthesis overview

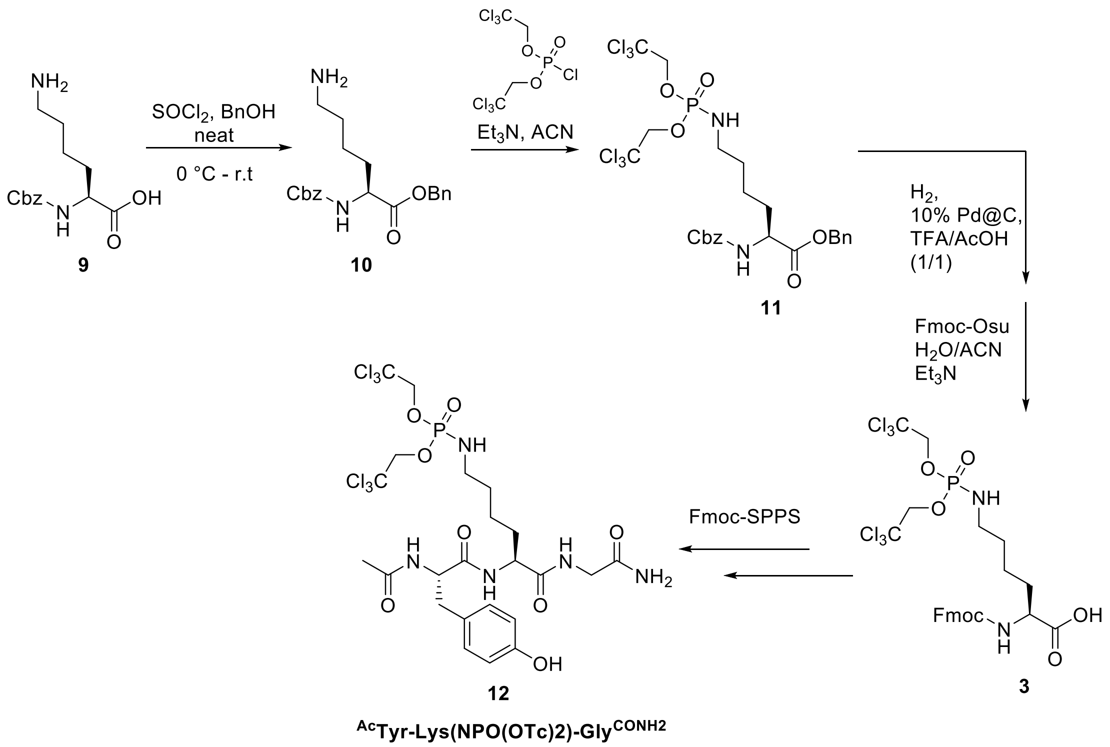

^{Ac}Tyr-Lys(NPO(OTc)2)-Gly^{CONH2}

3.4.1.2 Benzyl ((benzyloxy)carbonyl)-*L*-lysinate, *Z*-Lys-OBn (10)

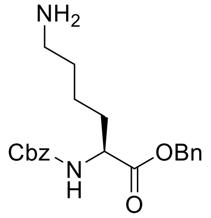

Benzyl protection of ((benzyloxy)carbonyl)-*L*-lysine was achieved by nucleophilic substitution. 1g (3.6 mmol) Z-Lys-OH was dissolved in benzyl alcohol to a 0.8 M solution and cooled in an ice bath for 5 min. 1.4 mL (18 mmol, 5 eq.) thionyl chloride were added dropwise to the mixture. After complete addition, the reaction was stirred 2 h at r.t. until UPLC analysis indicated complete conversion. The mixture was concentrated under reduced pressure and purified *via* silica column chromatography (100% EE+1% Et_3N → MeOH/EE 25/25+1% Et_3N, product eluting at 25% MeOH, silica filtered off after solvent evaporation). 1.20 g (3.2 mmol, 90%) product were obtained as colorless oil. ^{1}H NMR (600 MHz, CD_3CN) δ 7.43 – 7.25 (m, 10H), 6.27 (d, *J* = 8.1 Hz, 1H), 5.15 (q, *J* = 12.7 Hz, 2H), 5.08 (q, *J* = 12.6 Hz, 2H), 4.22 (td, *J* = 8.6, 4.9 Hz, 1H), 2.88 (t, *J* = 7.7 Hz, 2H), 1.85 – 1.65 (m, 2H), 1.63 (ddd, *J* = 21.0, 11.5, 4.3 Hz, 2H), 1.40 (qd, *J* = 9.2, 8.1, 5.1 Hz, 2H). ^{13}C NMR (151 MHz, CD_3CN) δ 175.01, 163.84, 163.61, 163.39, 163.16, 159.17, 158.56, 139.78, 138.81, 131.84, 131.80, 131.74, 131.55, 131.50, 131.46, 131.34, 131.29, 131.25, 131.04, 131.01, 130.95, 130.78, 130.73, 130.68, 130.44, 130.24, 130.17, 129.95, 129.92, 129.88, 122.56, 120.61, 118.67, 116.72, 70.31,69.98, 69.32, 69.00, 68.33, 68.02, 57.35, 56.41, 43.02, 42.07, 41.12, 34.22, 33.36, 32.51, 30.06, 29.22, 28.37, 25.82, 24.99, 24.14. R*f* (EE/MeOH 3/1+1% Et_3N) = 0.10. HR-MS for $C_{21}H_{26}N_2O_4$: *m*/*z* calc. $[M+H^+]^+$ = 371.1966, *m*/*z* obs. $[M+H^+]^+$ = 371.1971

3.4.1.3 Benzyl *N*2-((benzyloxy)carbonyl)-*N*6-(bis(2,2,2-trichloroethoxy)phosphoryl)-*L*-lysinate, *Z*-Lys(NPO(OTc)$_2$)-OBn (11)

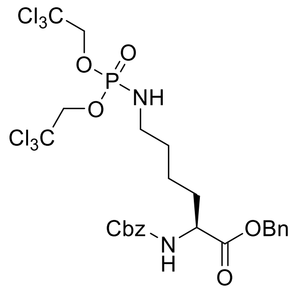

Compound **11** was synthesized in accordance to the previously described protocol by the Seebeck group.[17] Briefly, 1.20 g (3.24 mmol) **10** were dissolved to a 60 mM solution in ACN (54 mL) and 4 eq. Et_3N (13 mmol, 1.8 mL) were added and the pH checked for basicity. 1.47 g (3.88 mmol, 1.2 eq.) bis(2,2,2-trichloroethyl) phosphorochloridate were added in three equal portions of 491 mg each every 2 h while stirring at r.t. The reaction was kept at r.t. ovn and TLC indicated full conversion. Solvents were evaporated under reduced pressure and the crude product purified *via* silica column (hex/EE 8/2+0.25% FA → hex/EE 3/7+0.25% FA, product eluting at 40-

50% EE). Fractions containing **11** were concentrated under reduced pressure and residue formic acid removed by lyophilization. The product was obtained as a colourless oil (1.71 g, 2.40 mmol, 74%). ^{1}H NMR (600 MHz, CD_3CN) δ 7.43 – 7.26 (m, 10H), 6.08 (d, *J* = 8.1 Hz, 1H), 5.13 (q, *J* = 9.3 Hz, 2H), 5.07 (q, *J* = 8.1 Hz, 2H), 4.59 (dd, *J* = 6.2, 1.4 Hz, 4H), 4.24 – 4.15 (m, 1H), 3.92 (dt, *J* = 13.4, 6.9 Hz, 1H), 2.95 (dddd, *J* = 13.8, 11.8, 6.8, 1.2 Hz, 2H), 1.83 – 1.61 (m, 2H), 1.58 – 1.44 (m, 2H), 1.43 – 1.36 (m, 2H). ^{31}P NMR (243 MHz, CD_3CN) δ 4.52, 4.50, 4.48, 4.45, 4.43. ^{13}C NMR (151 MHz, CD_3CN) δ 175.07, 158.98, 139.85, 138.86, 131.82, 131.77, 131.72, 131.47, 131.27, 131.20, 130.95, 130.76, 130.71, 130.65,130.40, 130.21, 129.90, 98.14, 98.06, 79.79, 79.76, 78.75, 78.73, 77.72, 77.69, 70.17, 69.85, 69.19, 68.87, 68.20, 67.89, 57.43, 56.49, 44.39, 43.47, 42.55, 34.43, 34.12, 33.55, 33.28, 32.71, 32.46, 25.84, 25.03, 24.20. R*f* (hex/EE 7/3+0.25% FA) = 0.37. HR-MS for $C_{25}H_{29}Cl_6N_2O_7P$: *m*/*z* calc. $[M+H^+]^+$ = 710.9917, *m*/*z* obs. $[M+H^+]^+$ = 710.9915.

3.4.1.4 *N*6-(bis(2,2,2-trichloroethoxy)phosphoryl)-*L*-lysine, H_2N-Lys(NPO(OTc)$_2$)-OH

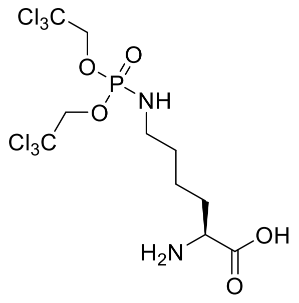

N- and *C*-terminal deprotection was achieved with hydrogenation. 600 mg (850 µmol) **11** were weighed into a Schlenk flask, dissolved to a 75 mM solution with AcOH:TFA:MeOH (5:5:90, v/v/v, 11 mL) and kept under vacuum for 5 min. In inert atmosphere, 96 mg Pd on activated charcoal (10% Pd, 113 mg per mmol starting material) were added and the gas exchanged with H_2. The mixture was stirred for 45 min in H_2 atmosphere until UPLC analysis indicated full conversion. The catalyst was filtered off and washed with MeOH. Solvents were evaporated by bubbling N_2 through the solution and residual acid removed by lyophilization. The crude product was applied in the next step without further purification.

3.4.1.5 *N*2-(((9*H*-fluoren-9-yl)methoxy)carbonyl)-*N*6-(bis(2,2,2-trichloroethoxy) phosphoryl)-*L*-lysine, Fmoc-Lys(NPO(OTc)$_2$)-OH (3)

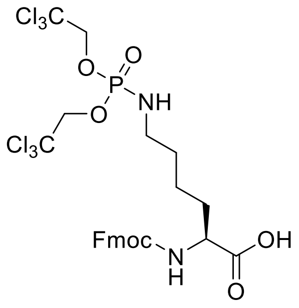

850 µmol crude product of H_2N-Lys(NPO(OTc)$_2$)-OH were dissolved in 2.1 mL H2O to a 0.4 M solution and 120 µL (850 µmol, 1 eq.) Et_3N were added. The pH was adjusted to 9 and a 0.4 M solution of 287 mg (850 µmol, 1 eq.) Fmoc-OSu in ACN (2.1 mL) was added while keeping the pH above 9 by further Et_3N addition. The reaction was allowed to proceed until the pH stabilized at pH 9.2. Subsequently, the pH was adjusted to 3.5 with AcOH and the mixture extracted 3x with $CHCl_3$. The combined organic layers were dried over Na_2SO_4 and solvents evaporated under reduced pressure. The crude product was purified *via* flash silica column (1. wash impurities off with hex/EE 45/55+0.25% FA, 2. elution of product with hex/EE 35/65+0.25% FA). After evaporation and lyophilization, 357 mg (502 µmol, 59%) product were obtained as white powder. ^{1}H NMR (600 MHz, CD_3CN) δ 7.83 (d, *J* = 7.7 Hz, 2H), 7.68 (t, *J* = 6.6 Hz, 2H), 7.42 (t, *J* = 7.6 Hz, 2H), 7.36 –7.31 (m, 2H), 6.07 (d, J = 8.0 Hz, 1H), 4.60 (d, J = 6.2 Hz, 4H), 4.33 (d, *J* = 7.2 Hz, 2H), 4.23 (t, *J* = 7.0 Hz, 1H), 4.13 – 4.05 (m, 1H), 3.94 (dt, *J* = 13.4, 6.9 Hz, 1H), 2.99 (dq, *J* = 13.4, 6.9 Hz, 2H), 1.84 – 1.62 (m, 1H), 1.59 – 1.47 (m, 2H), 1.43 (q, *J* = 8.2, 7.3 Hz, 2H). ^{31}P NMR (243 MHz, CD_3CN) δ 4.59, 4.57, 4.54, 4.52, 4.49, 4.46, 4.44, 4.41, 4.39. ^{13}C NMR (151 MHz, CD_3CN) δ 176.08, 158.93, 146.86, 143.86,130.95, 130.35, 129.89, 129.30, 123.27, 123.21, 122.15, 98.11, 79.77, 78.73, 77.70, 68.97, 57.03, 56.11, 50.21, 49.49, 49.34. R*f* (hex/EE: 1/1+0.25% FA) = 0.47. HR-MS for $C_{25}H_{27}Cl_6N_2O_7P$: *m*/*z* calc. $[M+H^+]^+$ = 708.9760, *m*/*z* obs. $[M+H^+]^+$ = 708.9764.

3.4.2 Peptide synthesis with building block 3 (peptide 12)

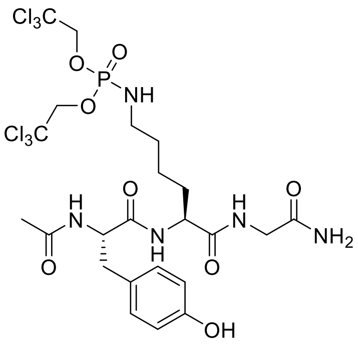

Peptide **12** was synthesized as described in section 2.2 on a 20 µmol scale with resin **17**. Incorporation of building block **3** was conducted as followed: 2.1 eq. AA (42 µmol, 30 mg) and 2 eq. HATU (40 µmol, 15 mg) were dissolved in 200 µL DMF to give a 0.2 M solution. Directly before adding the mixture to the Fmoc deprotected resin, 14 µL (4 eq., 80 µmol) DIPEA were added. The mixture was shaken at r.t. for 2 h, when the 2,4,6-trinitrobenzene-sulphonic acid (TNBS) test indicated full conversion. The peptide was capped before continuation of the peptide coupling. The crude product was purified on an analytical HPLC column to give 2.1 mg (2.8 µmol, 14% with regard to initial resin loading) white powder of desired peptide **12**. ^{1}H NMR (600 MHz, CD_3CN+10% D_2O) δ 7.14 (d, J = 8.5 Hz, 2H), 6.80 (d, J = 8.5 Hz, 2H), 4.69 (dd, J = 6.1, 1.7 Hz, 4H), 4.53 – 4.47 (m, 1H), 4.19 (dd, J = 9.2, 4.8 Hz, 1H), 3.78 (dd, J = 25.2, 17.0 Hz, 2H), 2.90 (dd, J = 14.0, 8.1 Hz, 2H), 1.95 (s, 3H), 1.88 – 1.81 (m, 2H), 1.66 (dtd, J = 14.2, 9.7, 5.1 Hz, 2H), 1.57 (ddt, J = 17.7, 9.1, 6.8 Hz, 2H), 1.40 – 1.32 (m, 2H). ^{31}P NMR (243MHz, CD_3CN+10% D_2O) δ 4.83, 4.81, 4.79, 4.76, 4.74, 4.71, 4.69. ^{13}C NMR (151 MHz, CD_3CN) *δ* 175.24, 174.98, 174.85, 174.24, 158.50, 133.13, 130.46, 117.84, 97.89, 78.80, 58.04, 56.40, 44.68, 43.38, 38.92, 33.28, 32.99, 25.03, 24.57. HR-MS for $C_{23}H_{32}Cl_6N_5O_8P$: *m*/*z* calc. $[M+H^+]^+$ = 748.0193, *m*/*z* obs. $[M+H^+]^+$ = 748.0192. t_R(gradient**I**) = 6.179min.

3.5 Synthesis of phospho-lysine peptide 8c via the Staudinger-phosphite reaction

3.5.1 Synthesis overview

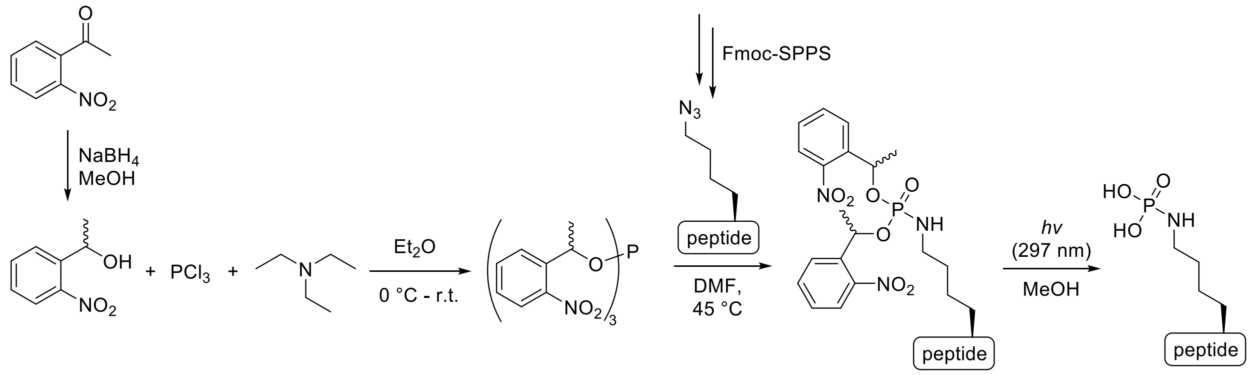

3.5.2 1-(2-nitrophenyl)ethanol (14)

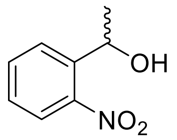

In a 500 mL round-bottom flask 10 g (60.6 mmol) 2'-nitroacteophenone were dissolved in 100 mL MeOH:dioxane (3:2, v/v) and cooled with an ice bath. Under vigorous stirring, 2.5 eq. sodium borohydride (151.4mmol, 5.7 g) were added portion wise over 90 min. The resulting mixture was equipped with septum and balloon and left to warm to r.t. ovn while stirring. After 16 h residual $NaBH_4$ was quenched by the addition of 50 mL of acetone and the solvents evaporated under reduced pressure. The residue was diluted with H_2O and EE and the layers separated. The organic layer was washed twice with water, the combined aqueous layers were washed once with EE, eventually, the combined organic layers were washed with brine, dried over $MgSO_4$ and the filtered solution concentrated under reduced pressure. The residual solvent was evaporated under high vacuum ovn. The product (9.7 g, 58.2 mmol, 96%) was obtained as a yellow oil. ^{1}H-NMR (600 MHz, CD_3CN) δ 7.86 (dd, J = 8.1, 1.4 Hz, 2H), 7.71 (td, J = 7.6, 1.3 Hz, 1H), 7.47 (ddd, J = 8.7, 7.4, 1.5 Hz, 1H), 5.30 (qd, J = 6.4, 4.1 Hz, 1H), 3.55 (dd, J = 4.2, 1.3 Hz, 1H), 1.48 (d, J = 6.4 Hz, 3H). ^{13}C-NMR (151 MHz, CD_3CN) δ 147.84, 141.58, 133.31, 127.91, 127.62, 123.75, 64.64, 24.19. HR-MS for $C_8H_9NO_3$: m/z calc. $[M+H^+]^+$ = 166.0499, m/z obs. $[M+H^+]^+$ = 166.0505.

3.5.3 Tris(1-(2-nitrophenyl)ethyl) phosphite (15)

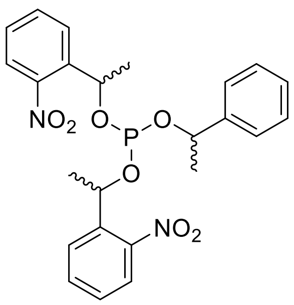

Phosphite **15** was synthesized by the condensation of trichlorophosphane and the alcohol **14** under inert conditions. A Schlenk flask was equipped with 1.7 g 1-(2 nitrophenyl)ethanol **14** (10.2 mmol, 3.4 eq.) and magnetic stir bar and set under high vacuum for 10 min. After balancing the pressure with argon, 10 mL dry THF were added and the solution cooled in an ice bath for 5 min. 1.33 mL Et_3N (9.6 mmol, 3.2 eq.) were added, the mixture left for another 2 min, while preparing a 0.6 M solution of 0.27 mL trichlorophosphane (3 mmol, 1.0 eq.) in dry THF (5 mL). The PCl_3 solution was added dropwise to the Schlenk flask, whereby a white precipitate was formed. After complete addition the mixture was kept in the ice bath for another 15 min, then stirred at r.t. under exclusion of light ovn. After 18 h the precipitate was filtered, washed with EE and the filtrate concentrated under reduced pressure. 1.03 g (2.19 mmol, 73%) product were obtained after column chromatography (hex/EE 9/1+1% Et_3N → hex/EE 7/3+1% Et_3N, the product eluting at 25% EE) as yellow oil and kept under argon, protected from light in the freezer until further usage. ^{1}H-NMR (300MHz, CD_3CN) δ 7.92 – 7.74 (m, 3H), 7.70 – 7.34 (m, 9H), 5.84 – 5.57 (m, 3H), 1.52 – 1.37 (m, 6H), 1.29 – 1.19 (m, 3H). ^{31}P-NMR (122 MHz, CD_3CN) δ 137.82, 135.97. ^{13}C-NMR (75 MHz, CD_3CN) δ 146.95, 146.86, 146.71, 146.44, 139.09, 139.04, 138.92, 138.89, 133.75, 133.73, 128.51, 128.42, 128.29, 128.02, 127.98, 127.90, 127.68, 124.10, 124.07, 124.06, 67.01, 66.83, 66.76, 66.64, 66.61, 66.50, 24.33, 24.28, 24.24, 24.20, 24.18, 24.13, 24.03, 23.98. R*f* (hex/EE 3/1+1% Et_3N) = 0.57.

3.5.4 ^{Ac}Tyr-Lys(N_3)-Gly^{CONH2} (16)

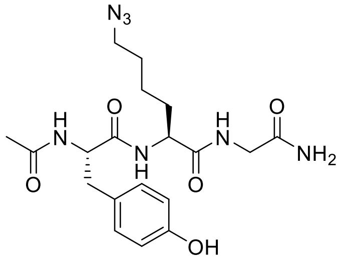

Peptide **16** was synthesized as described in section 2.2 on a 20 µmol scale with resin **17**. Incorporation of Fmoc-*az*-Lys-OH was conducted as described in section 2.2 with HATU as activating reagent. The mixture was shaken at r.t. for 2 h, when the TNBS test indicated full conversion. The peptide was capped before continuation of the peptide coupling. The crude product was purified on an analytical HPLC column to give 5.11 mg (11.8 µmol, 59% with regard to initial resin loading) white powder of desired peptide **16**. HR-MS for $C_{19}H_{27}N_7O_5$: *m*/*z* calc. $[M+H^+]^+$ = 433.2074, *m*/*z* obs. $[M+H^+]^+$ = 433.2079. t_R (gradient **II**) = 3.458 min.

3.5.5 ^{Ac}Tyr-Lys(NPO(ONPE)$_2$)-Gly^{CONH2} (13)

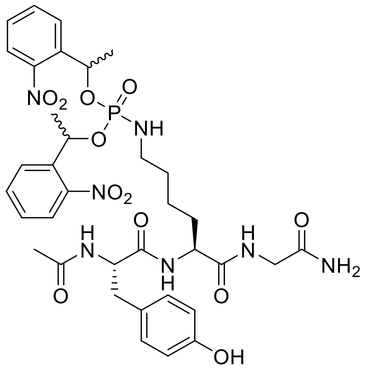

5.1 mg (11.8 µmol) azido-peptide were dissolved in 400 µL dry DMF. 18.7 mg (35.4 µmol, 3 eq.) phosphite **15** were dissolved in 72 µL dry DMF and added to the azide (final azide concentration 25 mM). The reaction was kept at 45 °C and samples taken for UPLC analysis at distinct time points. After 7 h and 26 h additional 2 eq. phosphite **15** were added to the reaction in order to drive it to completion. After 48 h, solvents were evaporated under reduced pressure and residual DMF removed during lyophilization. The product was obtained as a white powder after purification *via* analytical HPLC (7.0 mg, 9.0 µmol, 76%). HR-MS for $C_{35}H_{44}N_7O_{12}P$: *m*/*z* calc. $[M+H^+]^+$ = 786.2865, *m*/*z* obs. $[M+H^+]^+$= 786.2870. t_R (gradient **I**) = 6.212 min.

3.5.6 ^{Ac}Tyr-pLys-Gly^{CONH2} (8c)

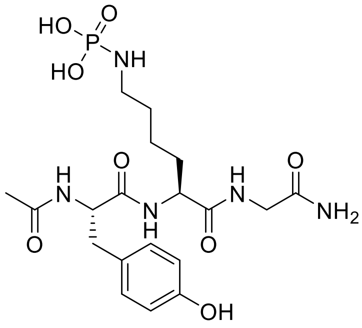

0.5 µmol caged-pLys peptide **14** (0.39 mg) was deprotected and worked up as described in the photodeprotection section 2.7. HR-MS for $C_{19}H_{30}N_5O_8P$: *m*/*z* calc. $[M+H^+]^+$ = 488.1911, *m*/*z* obs. $[M+H^+]^+$ = 488.1923. t_R (gradient **II**) = 1.537 min.

4. UPLC/UV Chromatograms at 220 nm

Peptide ^{Ac}Tyr-hCys(EtPO(OH)$_2$)-Gly^{CONH2} (**8a**)

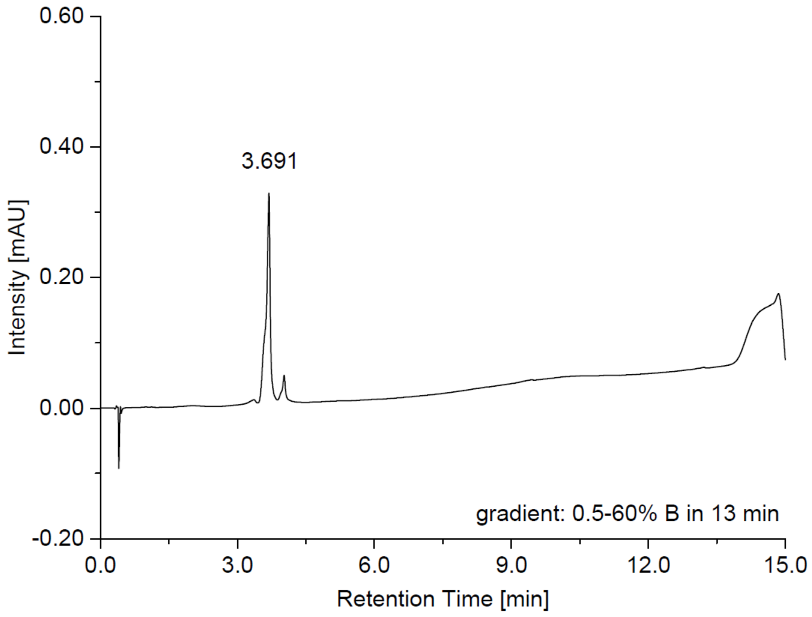

Peptide ^{Ac}Tyr-Nle(OPO(OH)$_2$)-Gly^{CONH2} (**8b**)

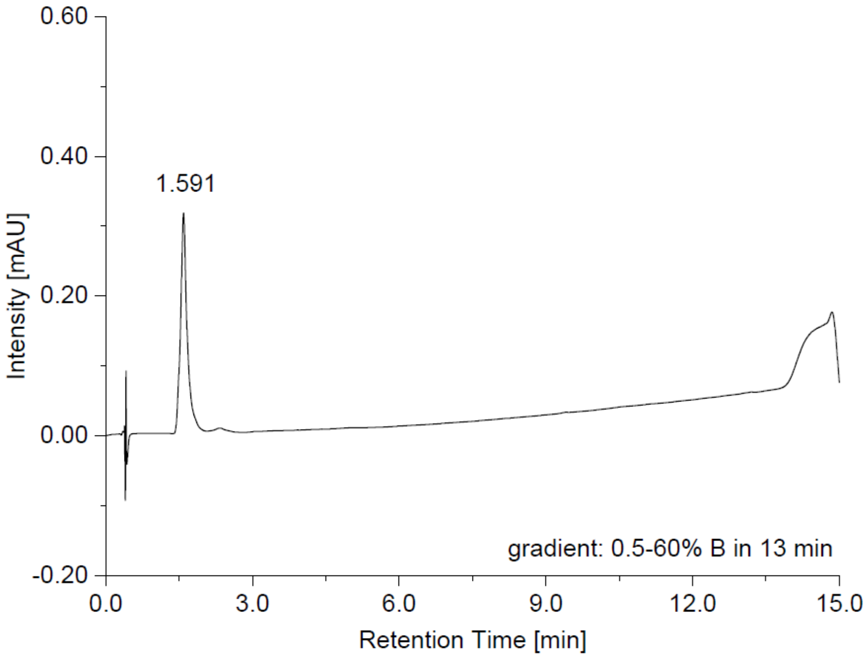

Peptide ^{Ac}Tyr-Lys(NPO(OTc)$_2$)-Gly^{CONH2} (**12**)

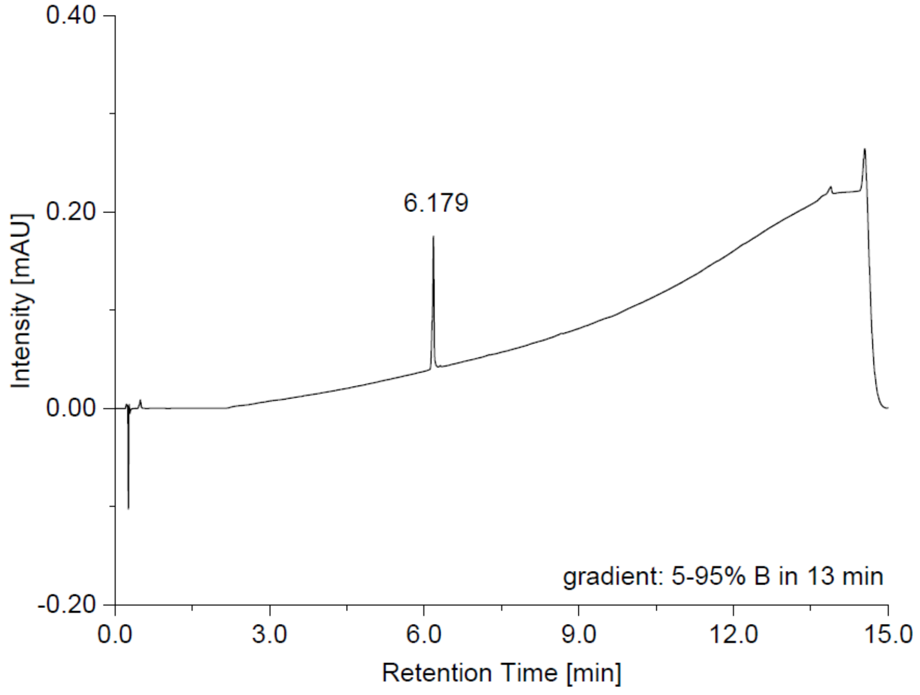

Peptide ^{Ac}Tyr-Lys(NPO(ONPE)$_2$)-Gly^{CONH2} (**13**)

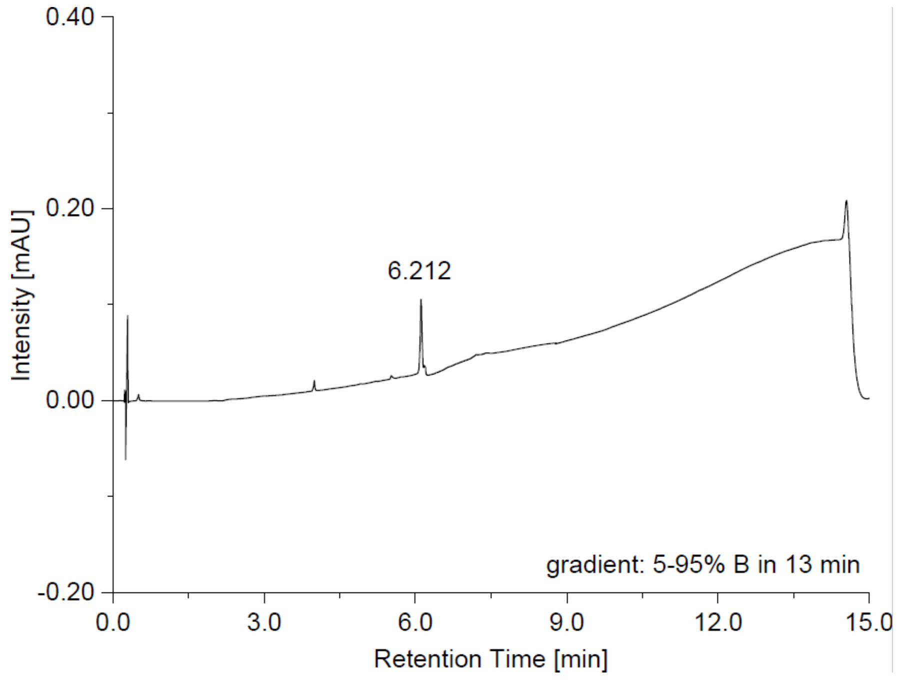

Peptide ^{Ac}Tyr-Lys(N_3)-Gly^{CONH2} **(16)**

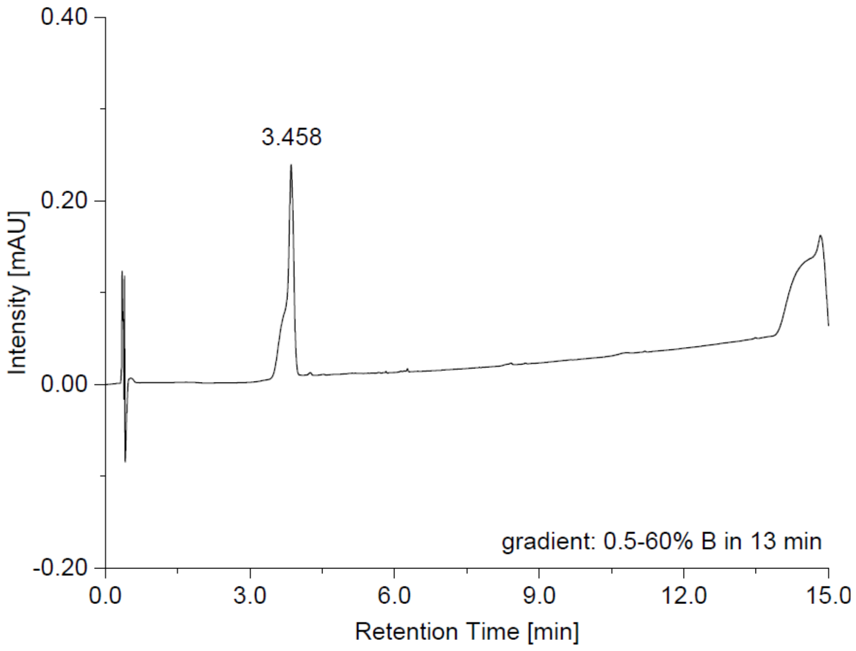

Peptide ^{Ac}Tyr-pLys-Gly^{CONH2} **(8c)**

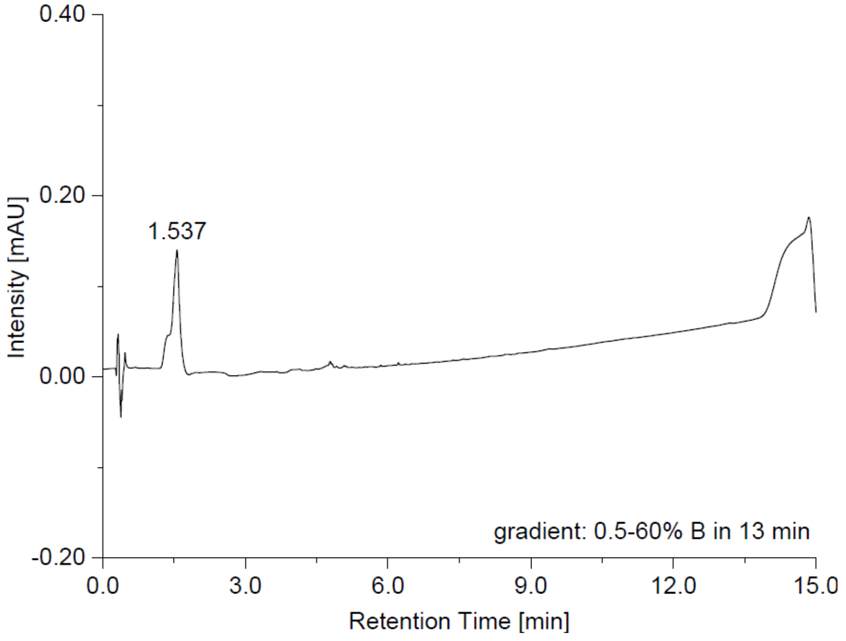

5. NMR Spectra

Vinylphosphonic dichloride (crude)

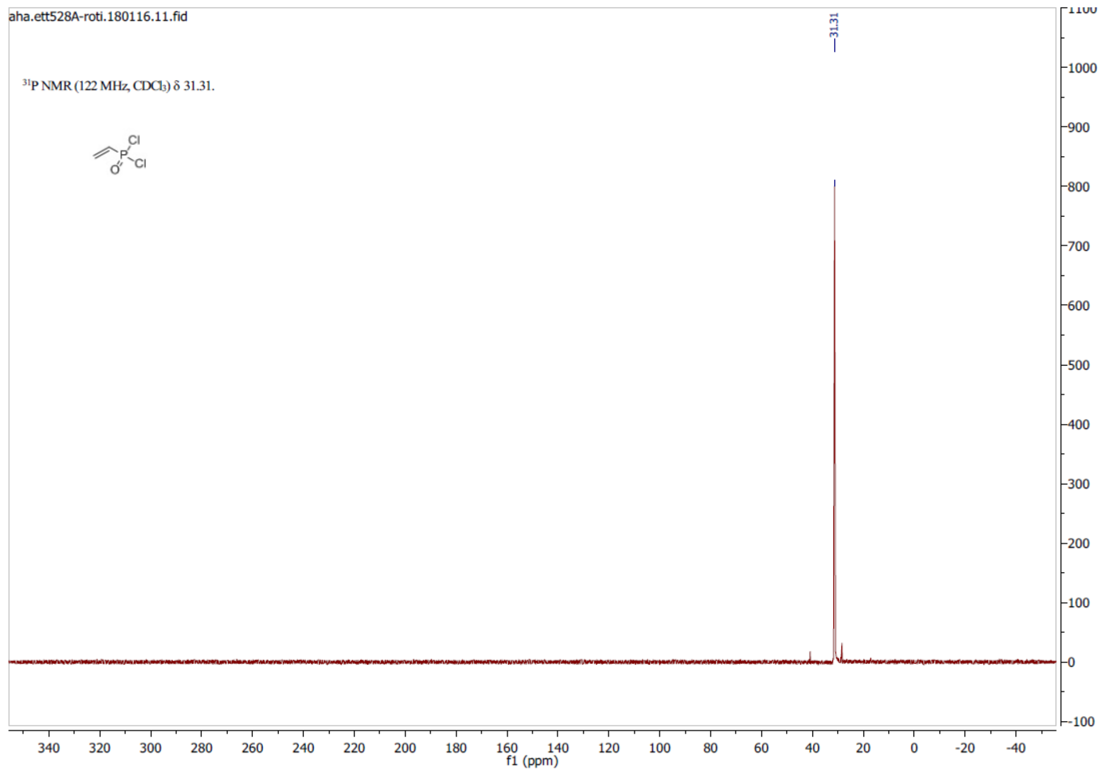

Dibenzyl vinylphosphonate (**4**)

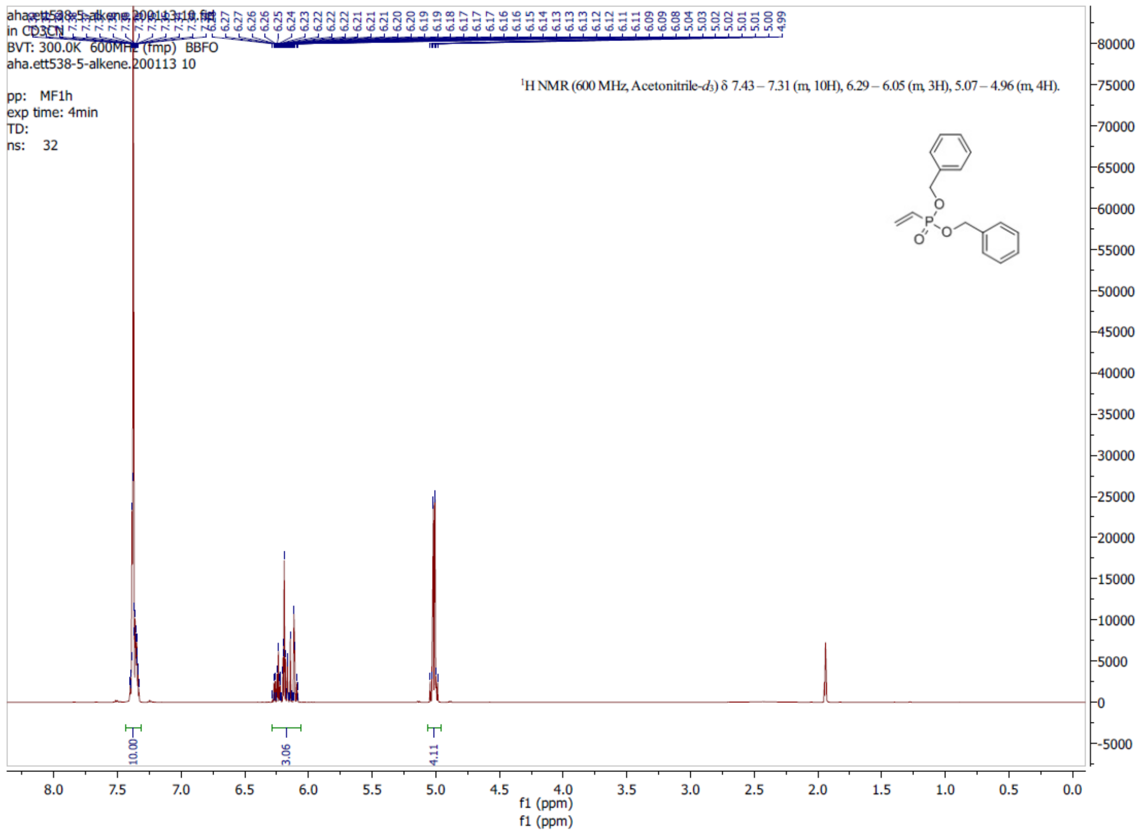

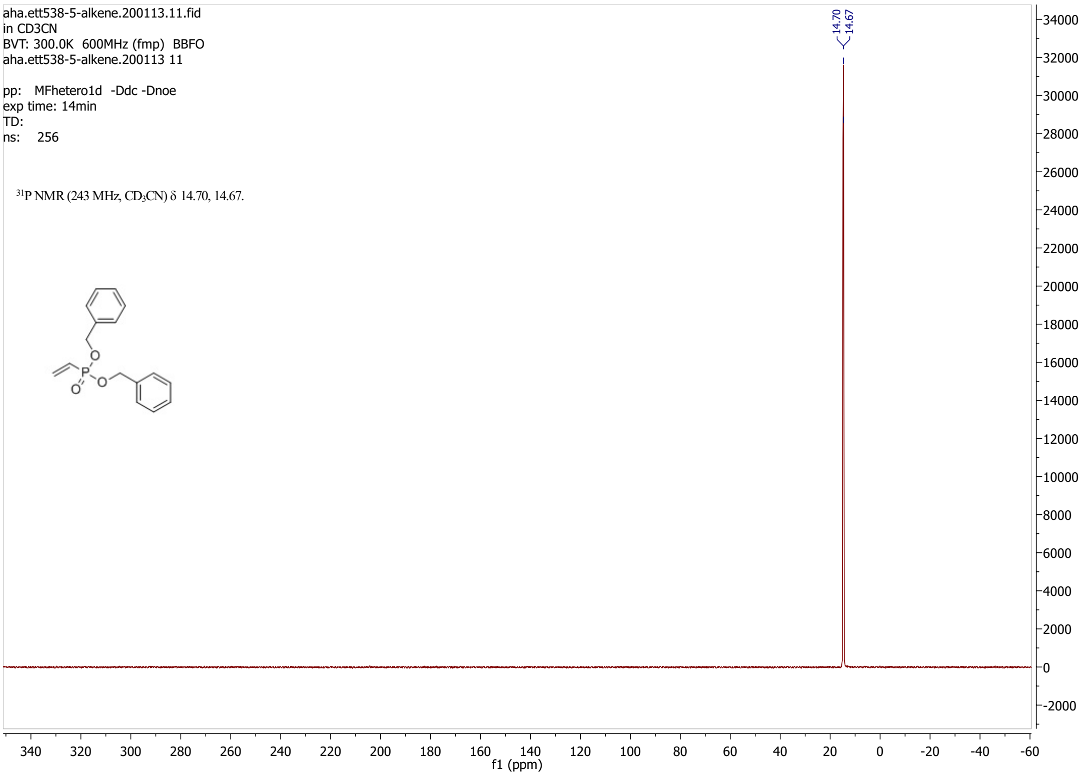
aha.ett538-5-alkene.200113.11.fid
in CD3CN
BVT: 300.0K 600MHz (fmp) BBFO
aha.ett538-5-alkene.200113 11
pp: MFhetero1d -Ddc -Dnoe
exp time: 14min
TD:
ns: 256
^{31}P NMR (243 MHz, CD_3CN) δ 14.70, 14.67.
14.70
14.67
f1 (ppm)

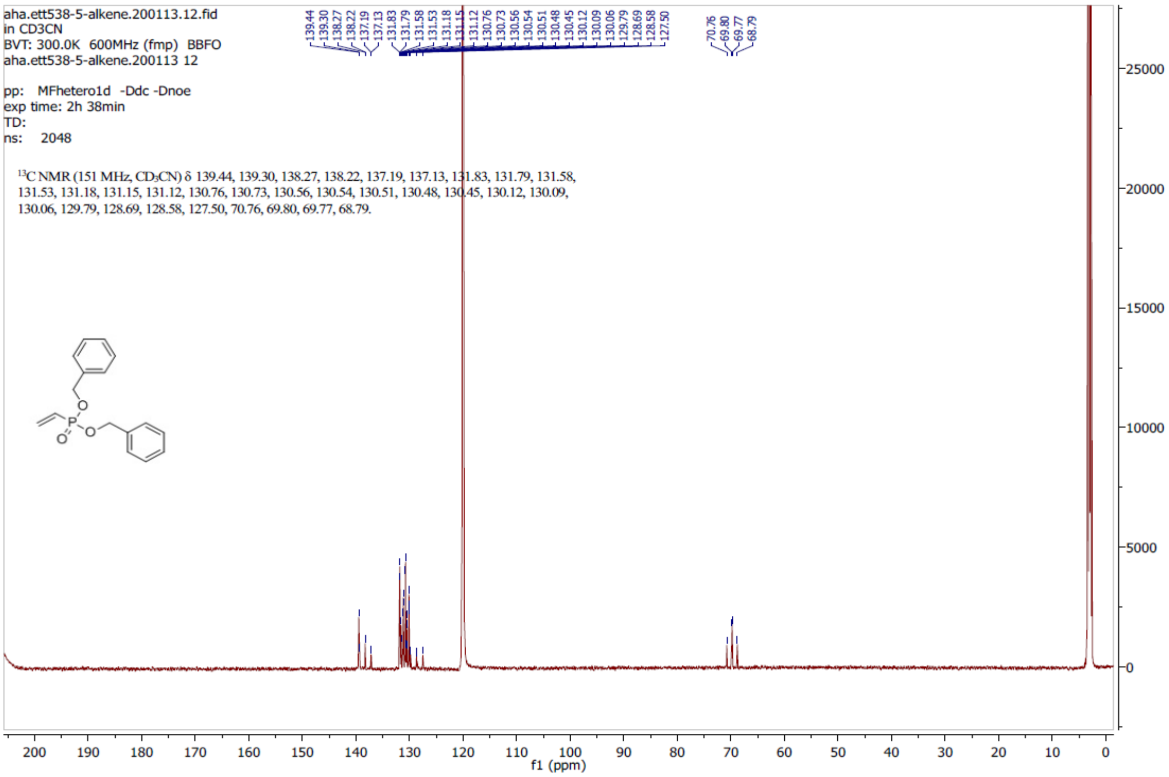
aha.ett538-5-alkene.200113.12.fid
in CD3CN
BVT: 300.0K 600MHz (fmp) BBFO
aha.ett538-5-alkene.200113 12
pp: MFhetero1d -Ddc -Dnoe
exp time: 2h 38min
TD:
ns: 2048
^{13}C NMR (151 MHz, CD_3CN) δ 139.44, 139.30, 138.27, 138.22, 137.19, 137.13, 131.83, 131.79, 131.58, 131.53, 131.18, 131.15, 131.12, 130.76, 130.73, 130.56, 130.54, 130.51, 130.48, 130.45, 130.12, 130.09, 130.06, 129.79, 128.69, 128.58, 127.50, 70.76, 69.80, 69.77, 68.79.
f1 (ppm)

(((9*H*-fluoren-9-yl)methoxy)carbonyl)-*L*-homocysteine (**5**)

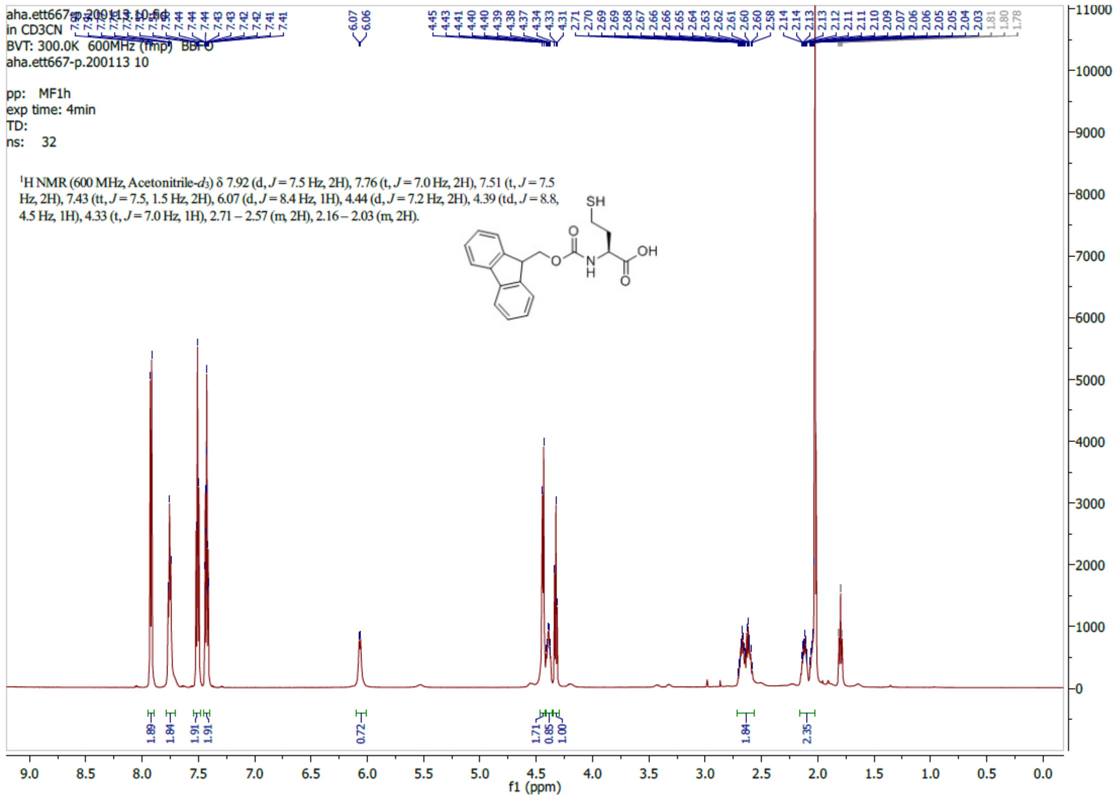

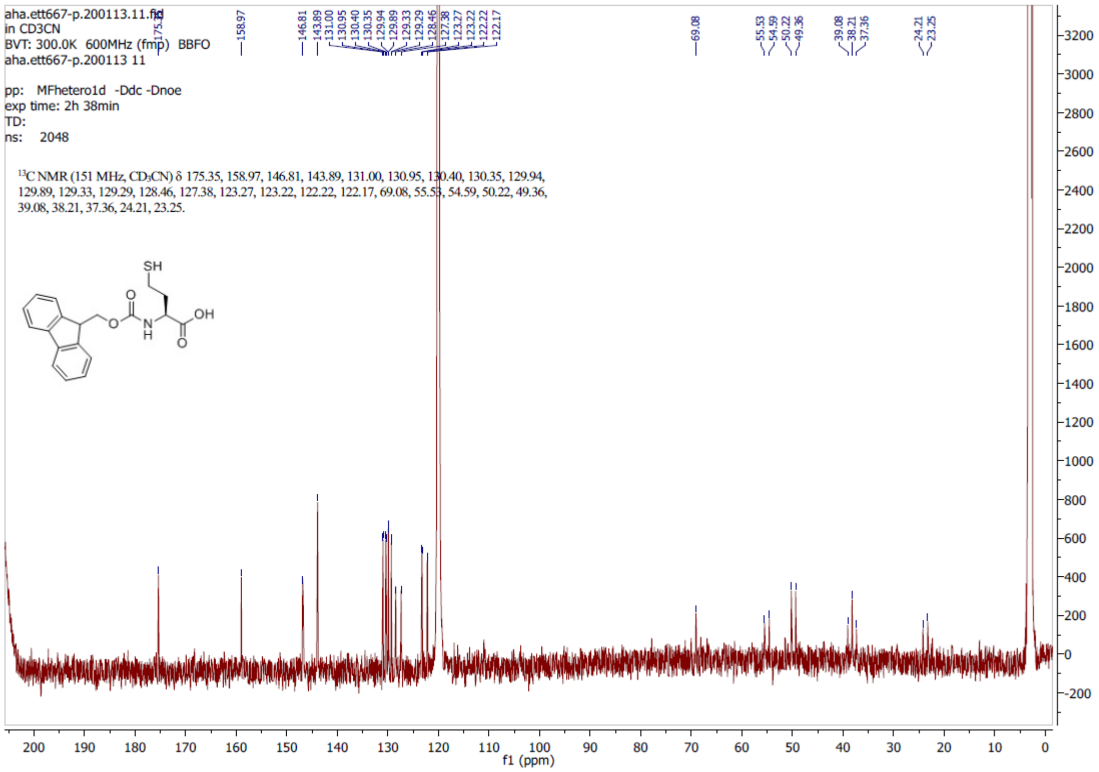

N-(((9*H*-fluoren-9-yl)methoxy)carbonyl)-*S*-(2-(bis(benzyloxy)phosphoryl)ethyl)-*L*-homocysteine (**1**)

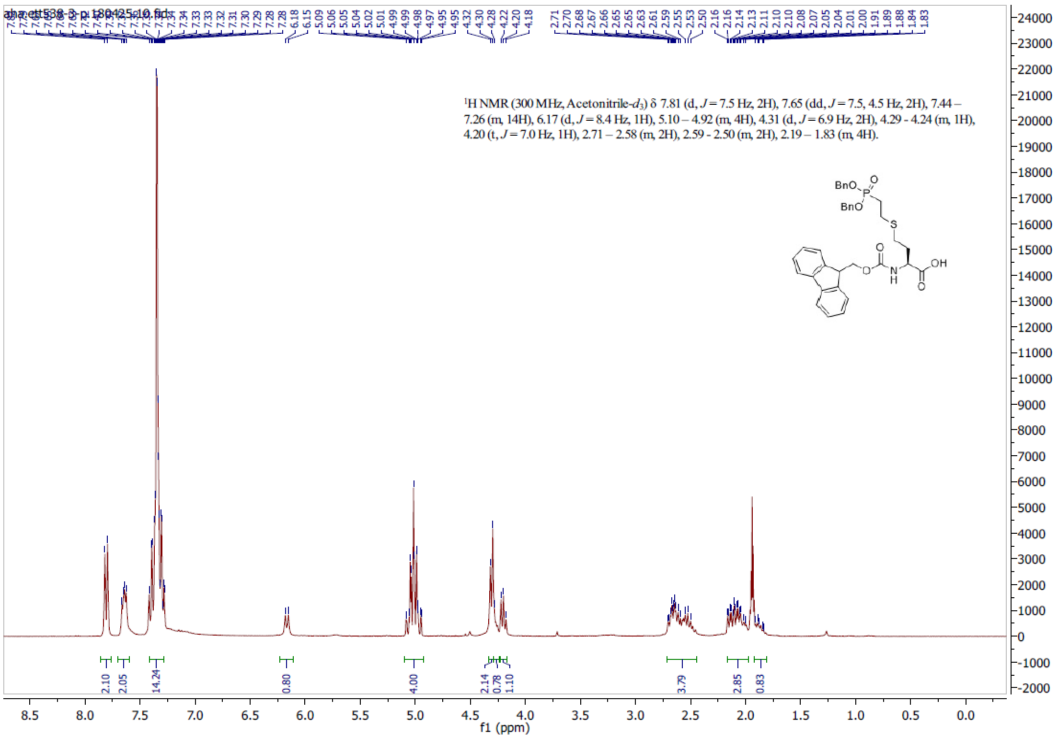

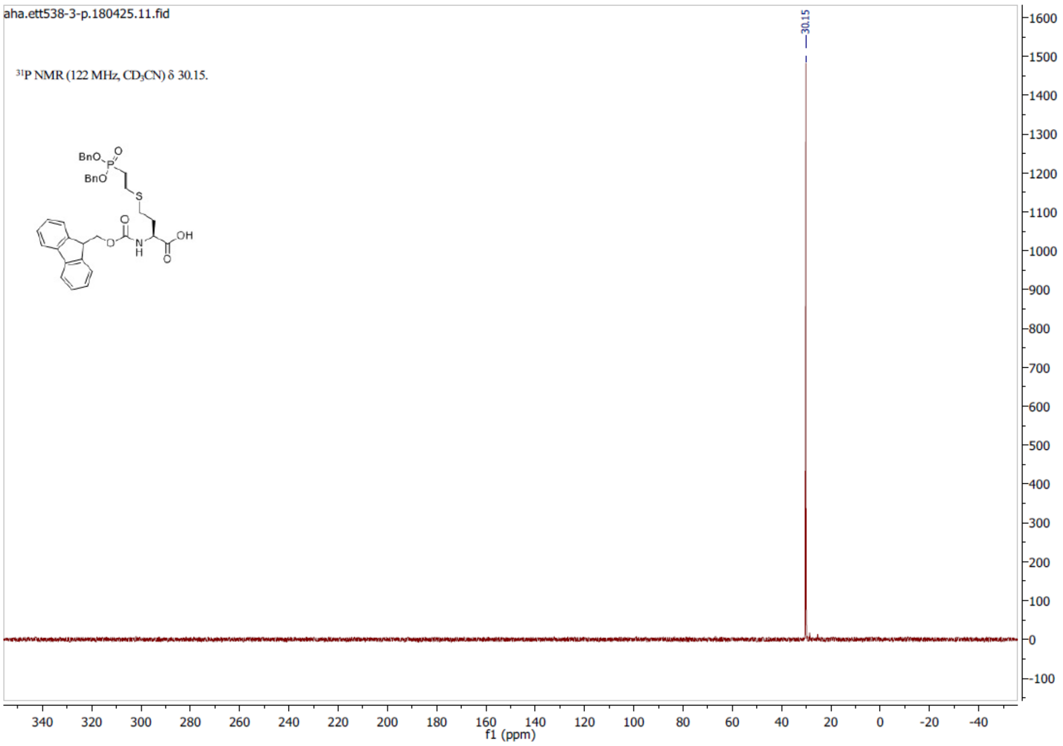
aha.ett538-3-p.180425.11.fid
31P NMR (122 MHz, CD3CN) δ 30.15.
30.15
BnO
BnO
P
O
S
O
N
H
OH
O
f1 (ppm)

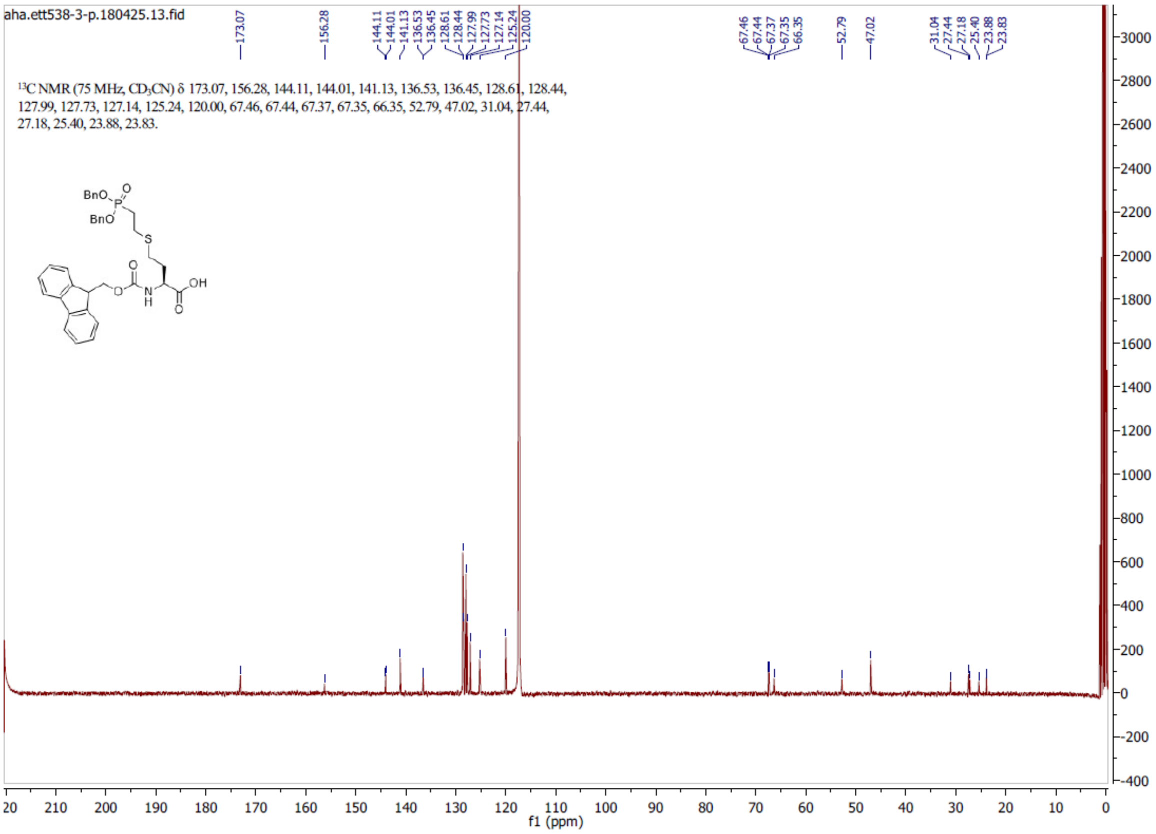
aha.ett538-3-p.180425.13.fid
13C NMR (75 MHz, CD3CN) δ 173.07, 156.28, 144.11, 144.01, 141.13, 136.53, 136.45, 128.61, 128.44, 127.99, 127.73, 127.14, 125.24, 120.00, 67.46, 67.44, 67.37, 67.35, 66.35, 52.79, 47.02, 31.04, 27.44, 27.18, 25.40, 23.88, 23.83.
BnO
BnO
P
O
S
O
N
H
OH
O
f1 (ppm)

(2*S*)-2-((((9*H*-fluoren-9-yl)methoxy)carbonyl)amino)-6-(((benzyloxy)(hydroxy)phosphoryl)oxy) hexanoic acid **(2)**

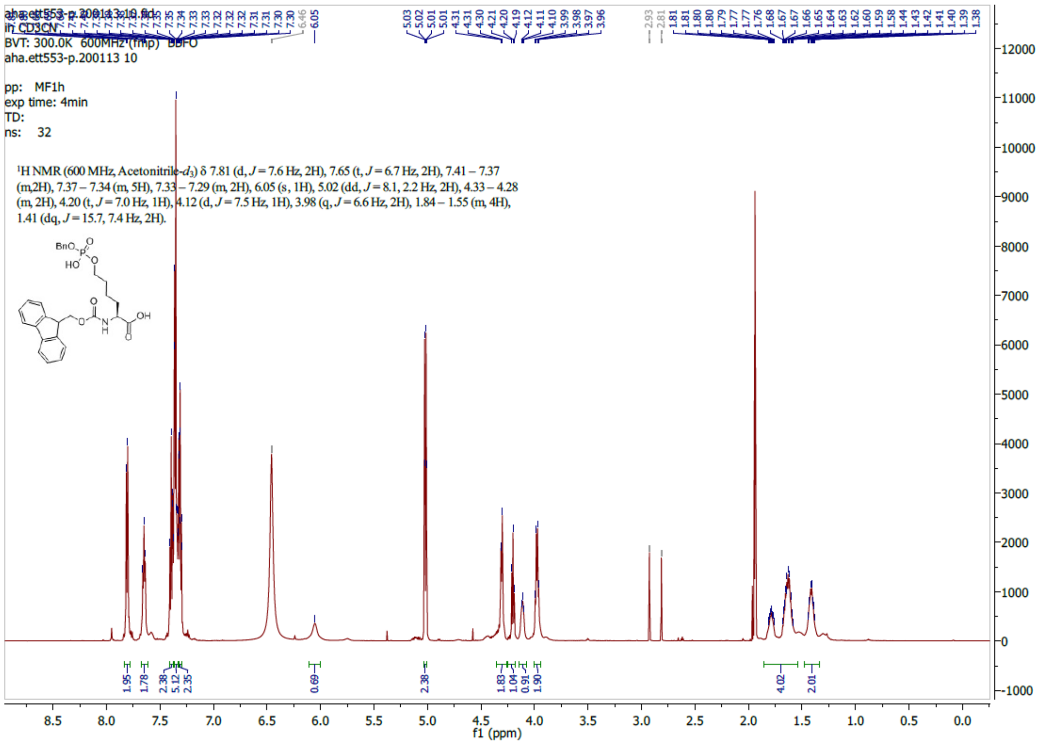

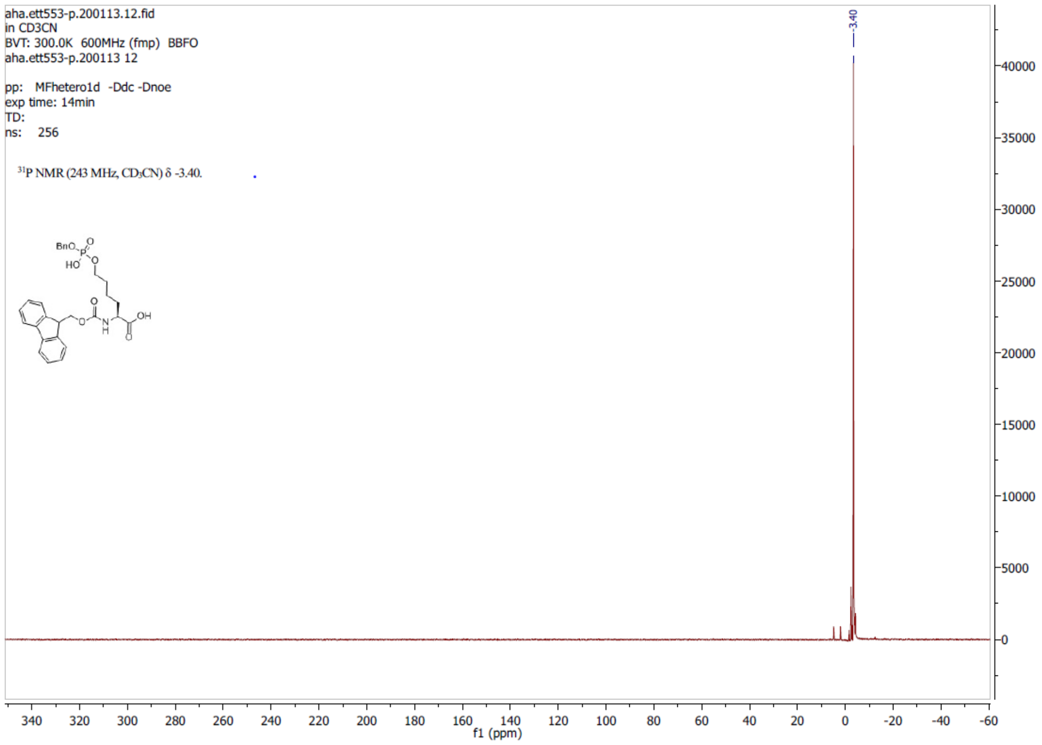

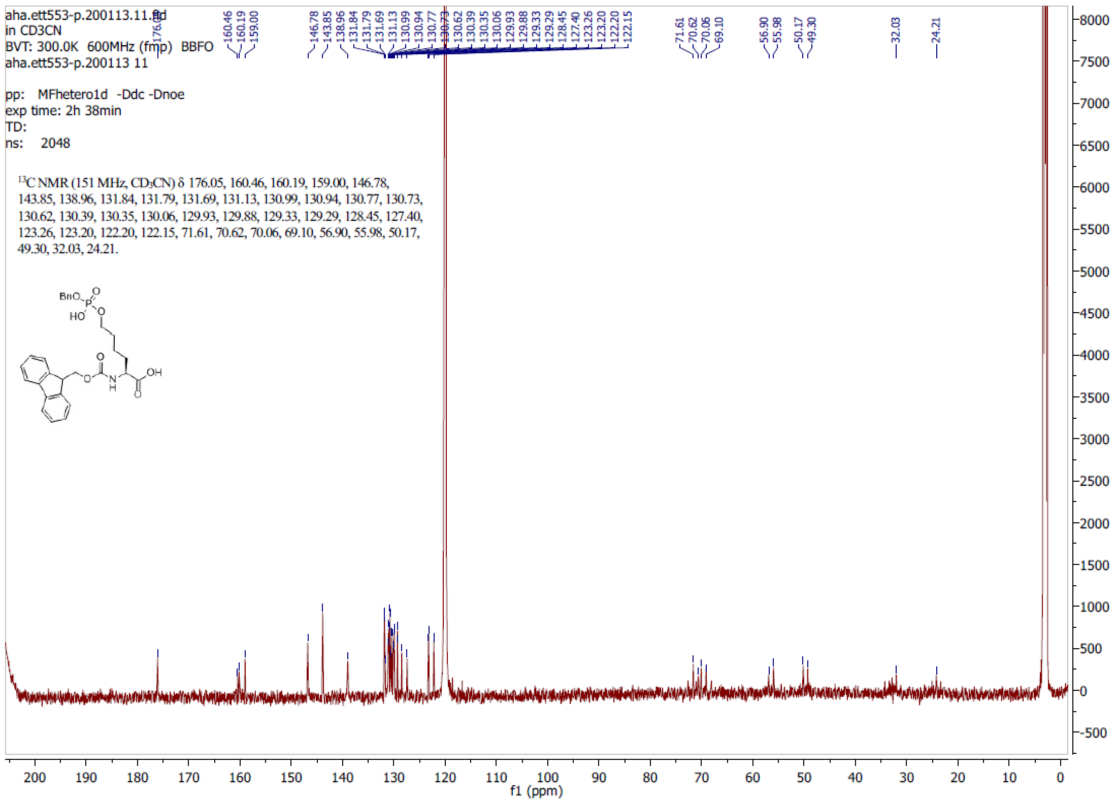

^{Ac}Tyr-Nle(OPO(OH)$_2$)-Gly^{CONH2} (**8b**)

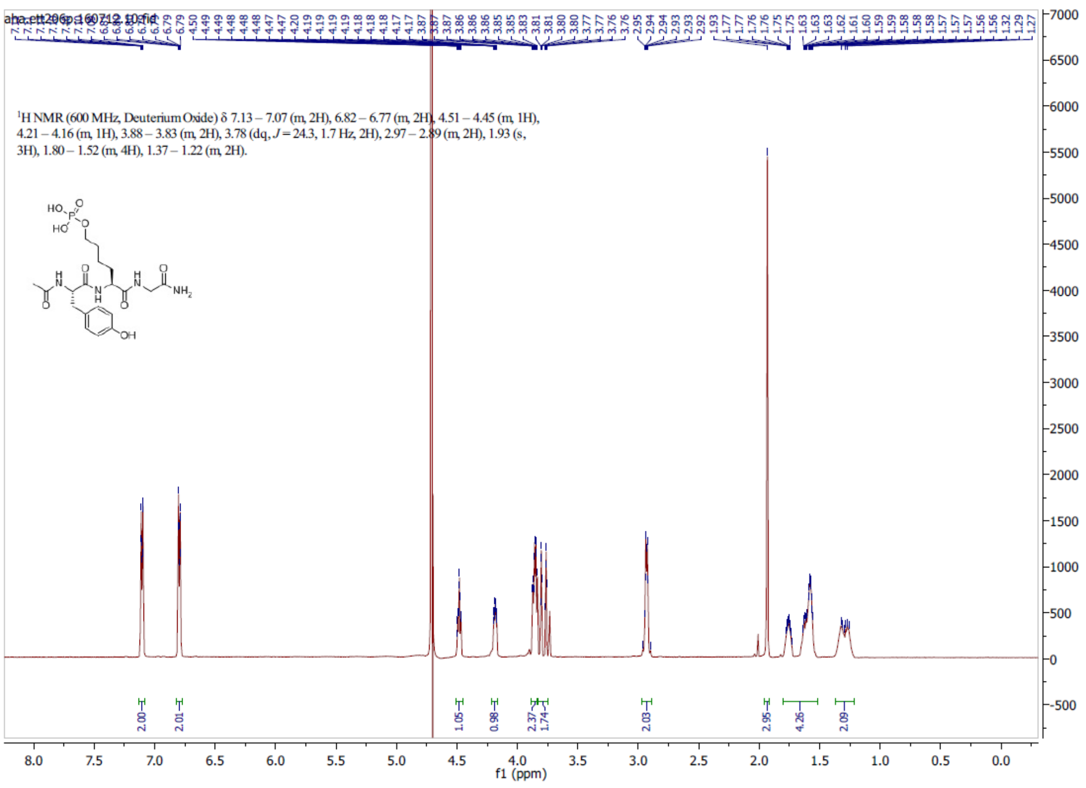

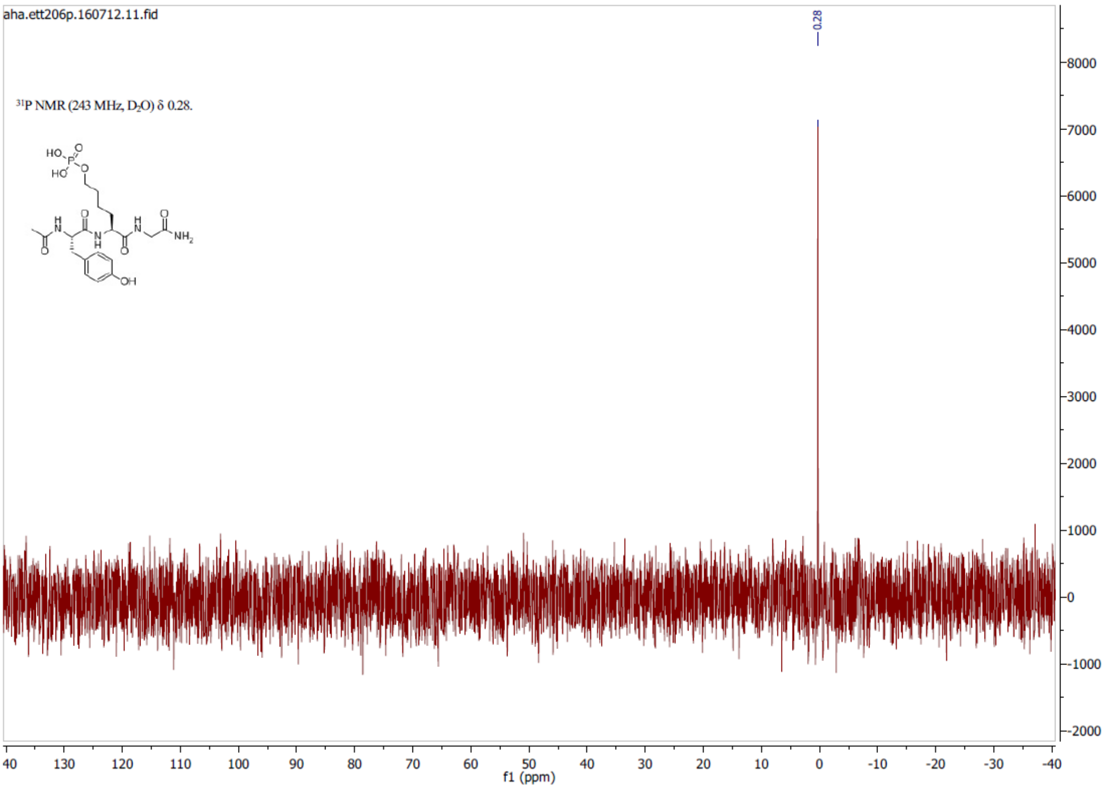
aha.ett206p.160712.11.fid
0.28
^{31}P NMR (243 MHz, D_2O) δ 0.28.
f1 (ppm)

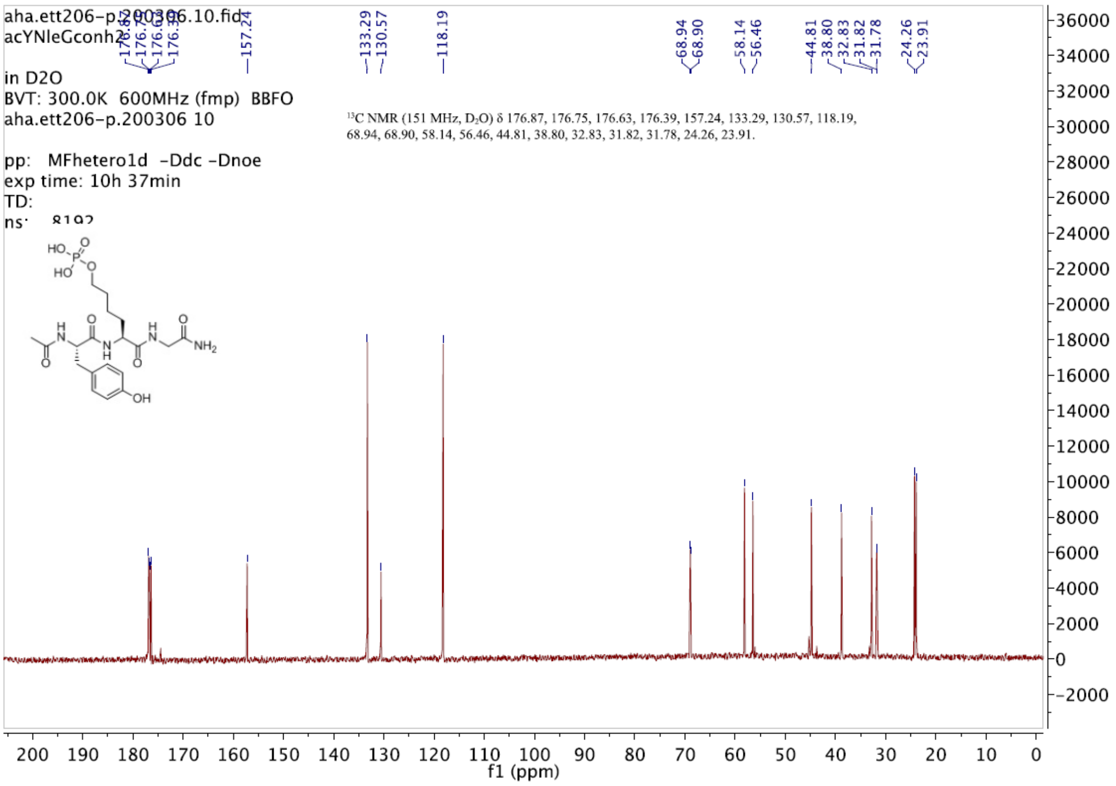
aha.ett206-p.200306.10.fid
acYNleGconh2
in D2O
BVT: 300.0K 600MHz (fmp) BBFO
aha.ett206-p.200306 10
pp: MFhetero1d -Ddc -Dnoe
exp time: 10h 37min
TD:
^{13}C NMR (151 MHz, D_2O) δ 176.87, 176.75, 176.63, 176.39, 157.24, 133.29, 130.57, 118.19, 68.94, 68.90, 58.14, 56.46, 44.81, 38.80, 32.83, 31.82, 31.78, 24.26, 23.91.
f1 (ppm)

Benzyl ((benzyloxy)carbonyl)-*L*-lysinate (**10**)

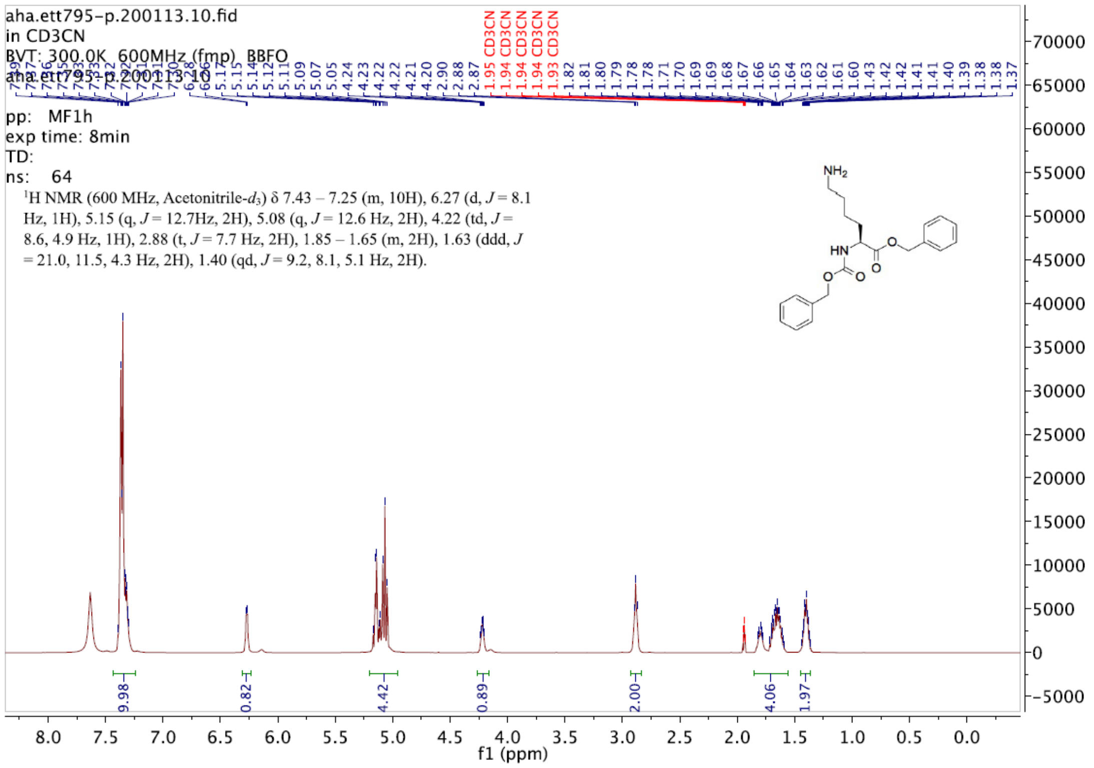

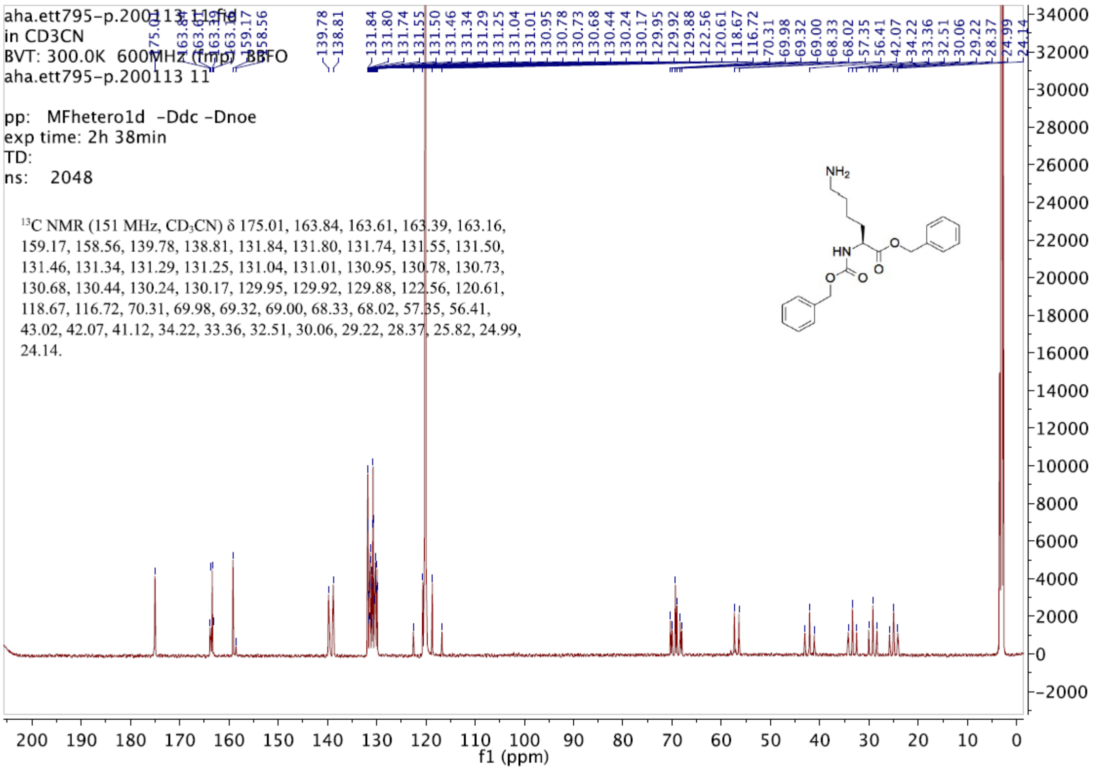

Benzyl *N*2-((benzyloxy)carbonyl)-*N*6-(bis(2,2,2-trichloroethoxy)phosphoryl)-*L*-lysinate (**11**)

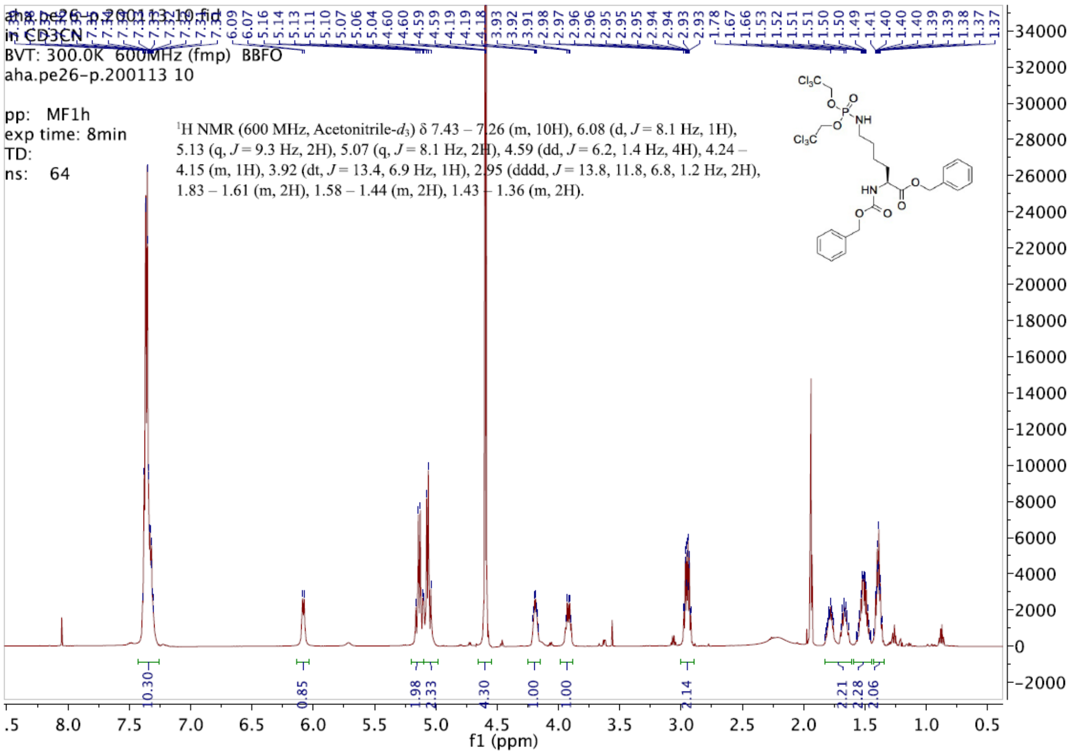

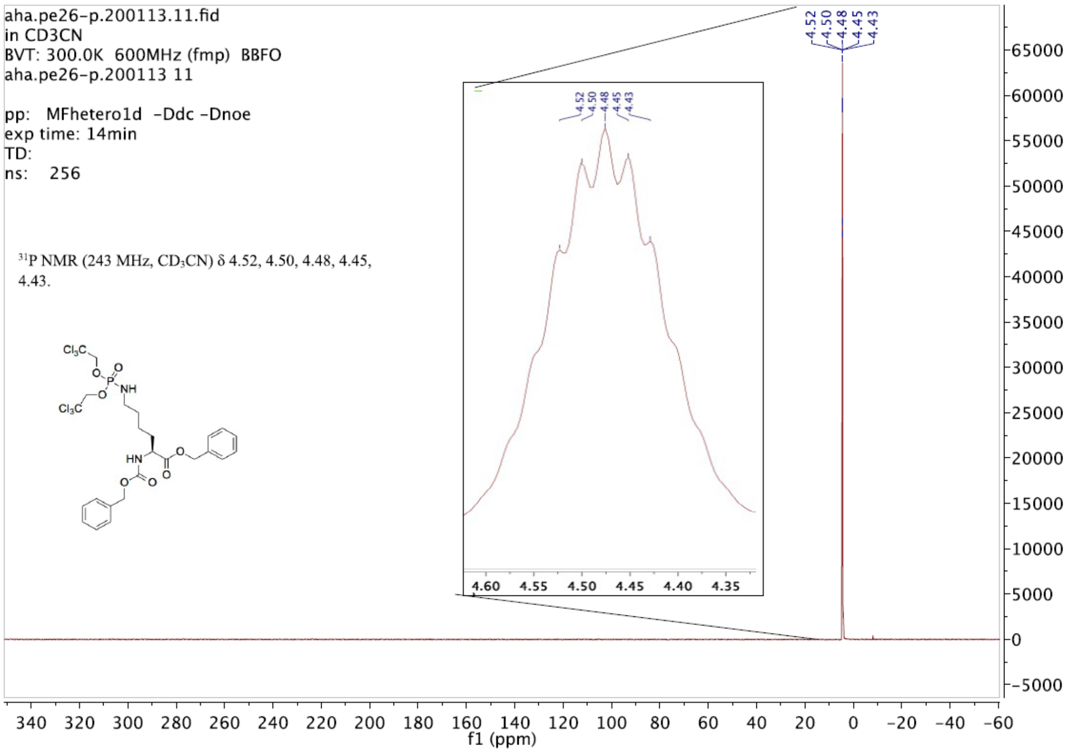

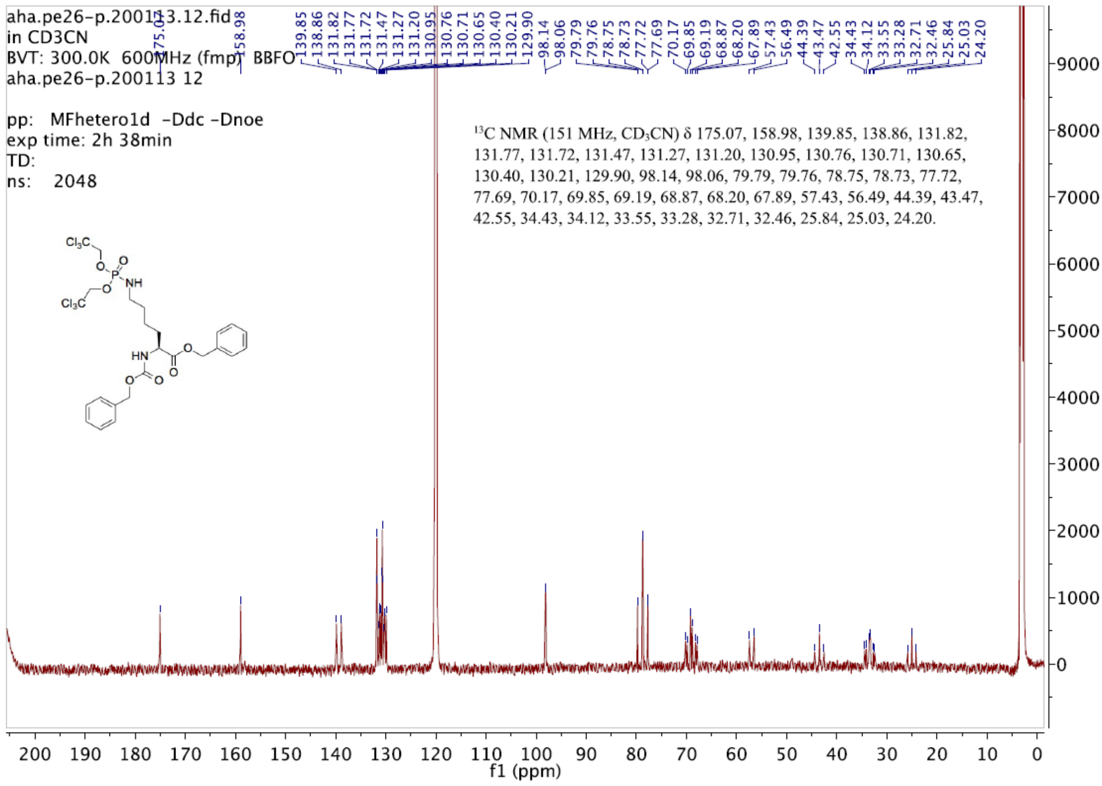

*N*2-(((9*H*-fluoren-9-yl)methoxy)carbonyl)-*N*6-(bis(2,2,2-trichloroethoxy) phosphoryl)-*L*-lysine (**3**)

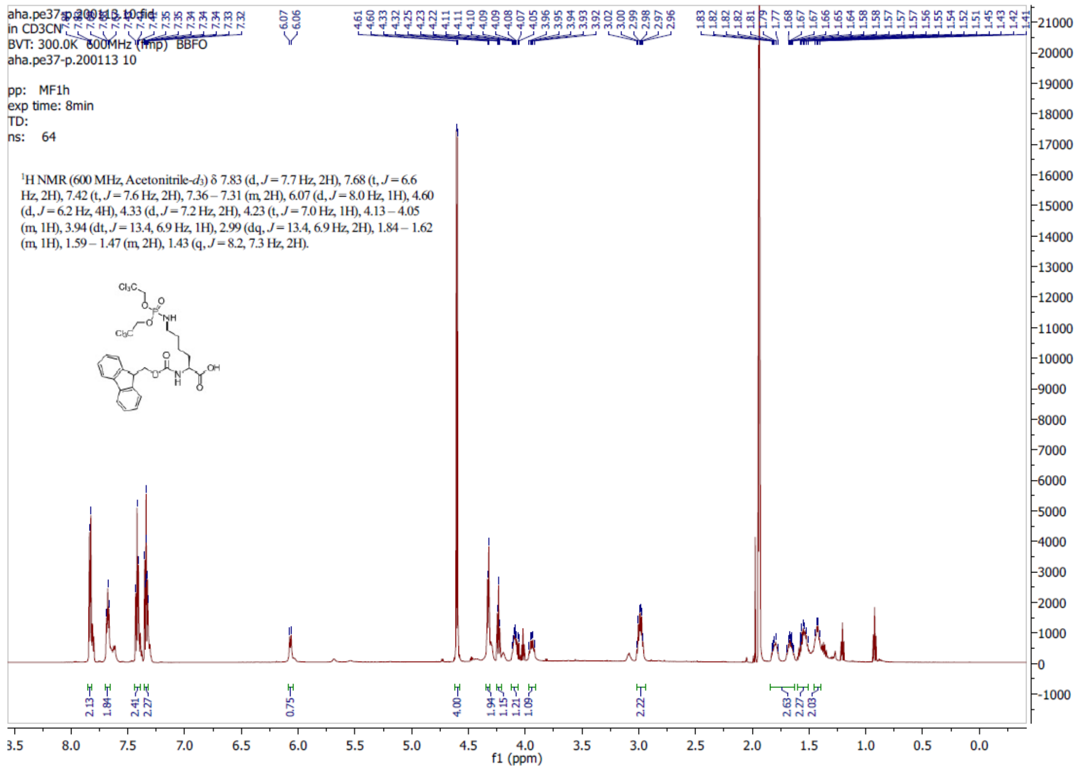

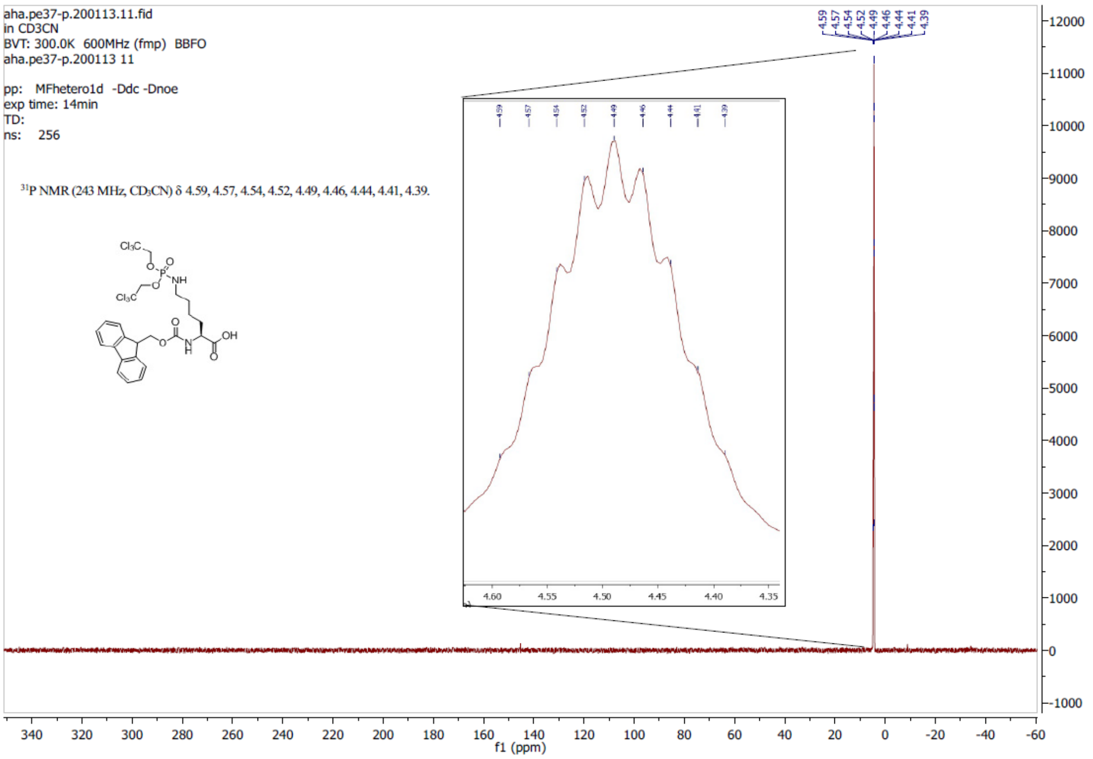
aha.pe37-p.200113.11.fid
in CD3CN
BVT: 300.0K 600MHz (fmp) BBFO
aha.pe37-p.200113 11
pp: MFhetero1d -Ddc -Dnoe
exp time: 14min
TD:
ns: 256
³¹P NMR (243 MHz, CD₃CN) δ 4.59, 4.57, 4.54, 4.52, 4.49, 4.46, 4.44, 4.41, 4.39.
f1 (ppm)

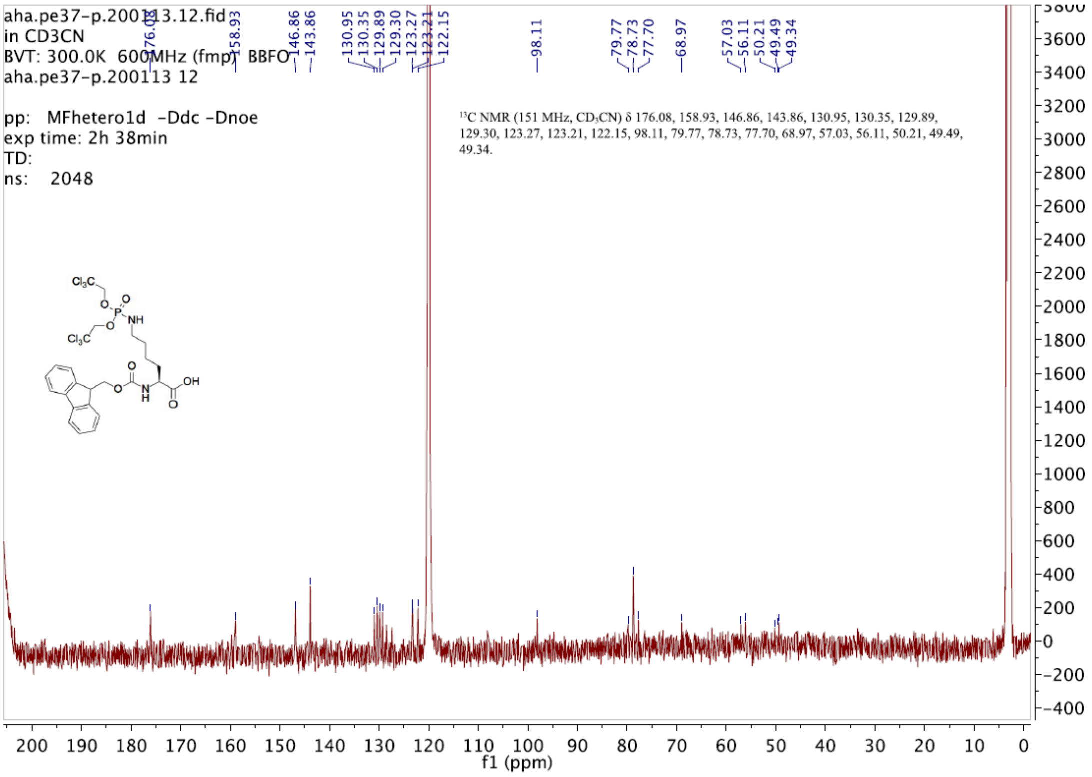
aha.pe37-p.200113.12.fid
in CD3CN
BVT: 300.0K 600MHz (fmp) BBFO
aha.pe37-p.200113 12
pp: MFhetero1d -Ddc -Dnoe
exp time: 2h 38min
TD:
ns: 2048
¹³C NMR (151 MHz, CD₃CN) δ 176.08, 158.93, 146.86, 143.86, 130.95, 130.35, 129.89, 129.30, 123.27, 123.21, 122.15, 98.11, 79.77, 78.73, 77.70, 68.97, 57.03, 56.11, 50.21, 49.49, 49.34.
f1 (ppm)

^{Ac}Tyr-Lys(NPO(OTc)2)-Gly^{CONH2} (**12**)

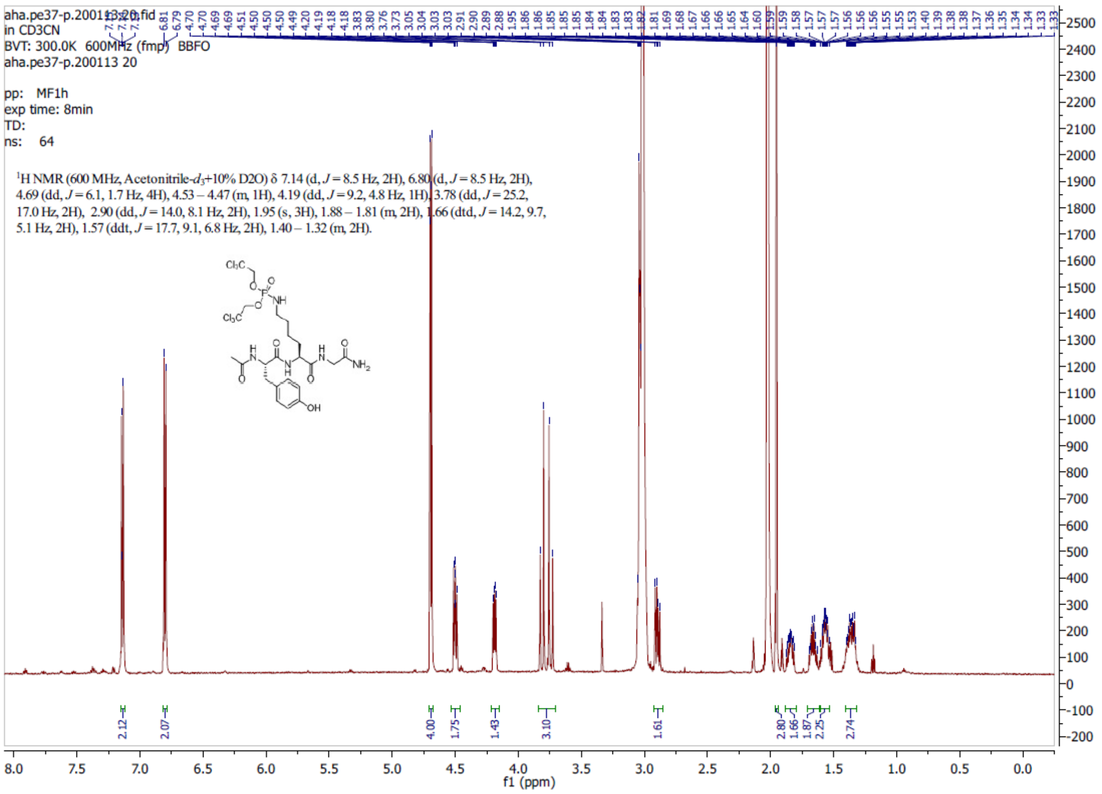

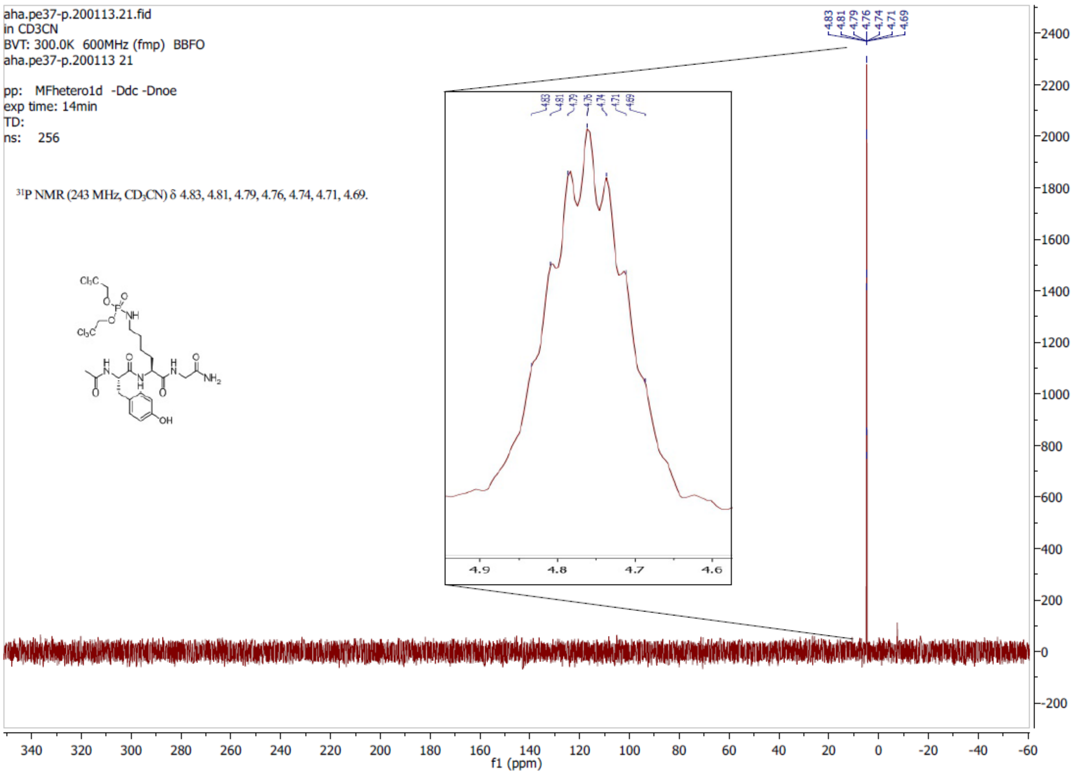

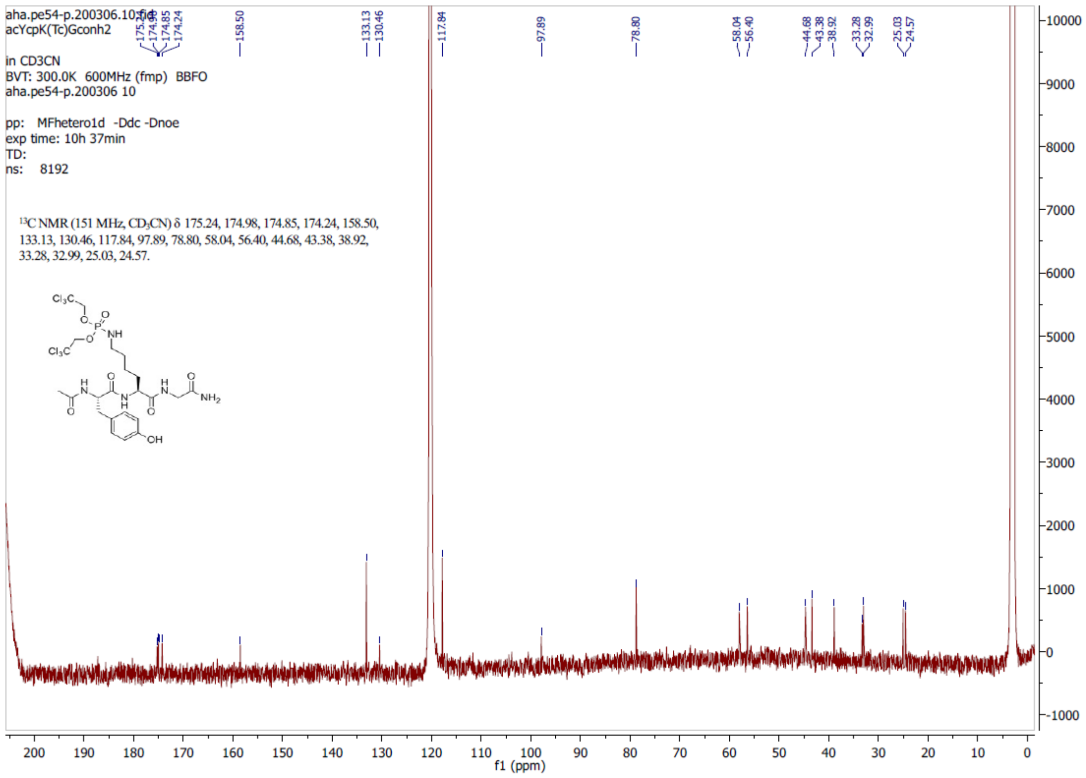

1-(2-nitrophenyl)ethanol (**14**)

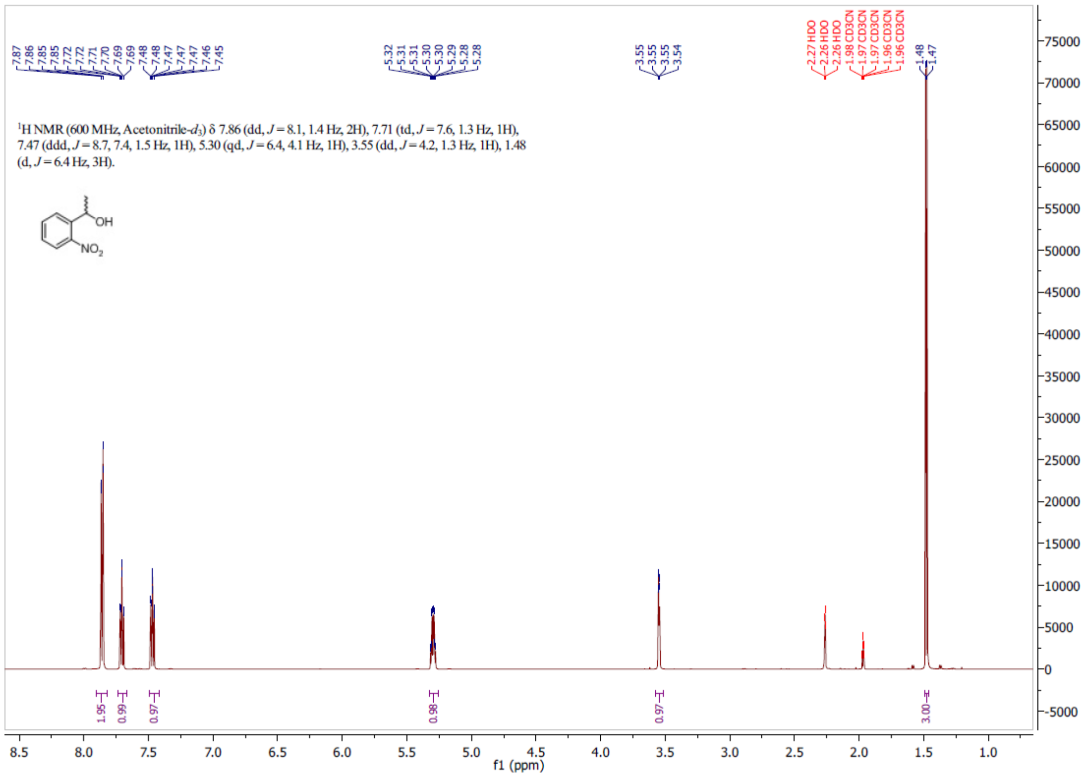

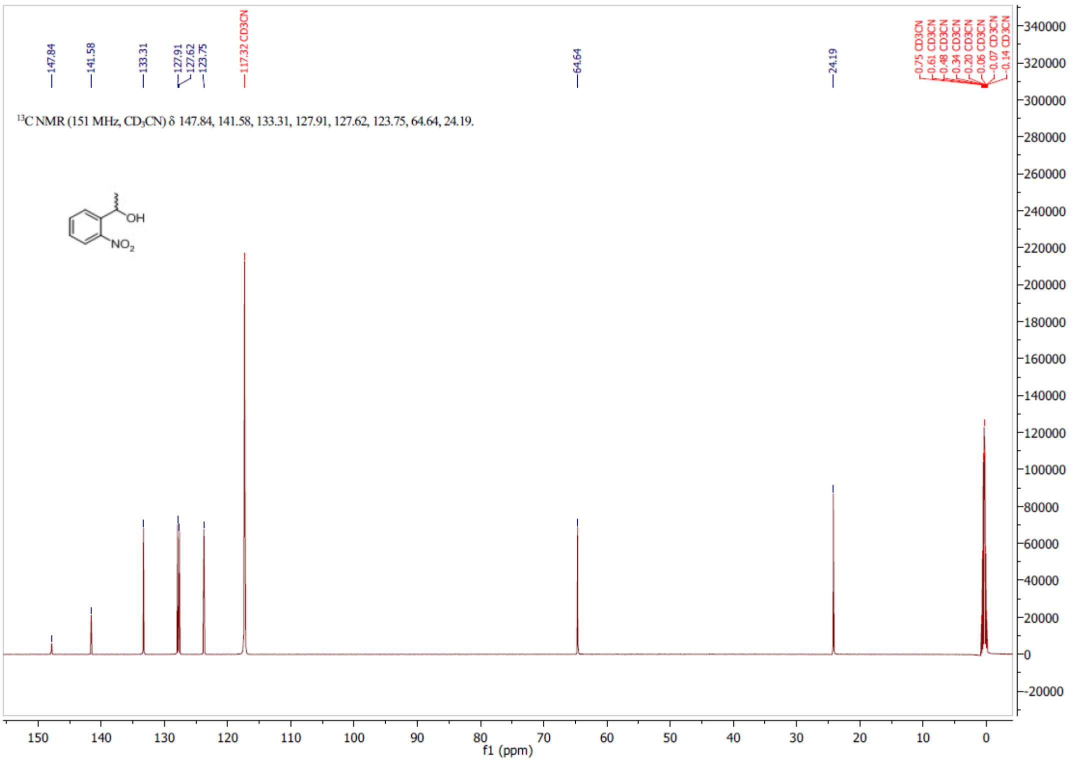

Tris(1-(2-nitrophenyl)ethyl) phosphite (**15**)

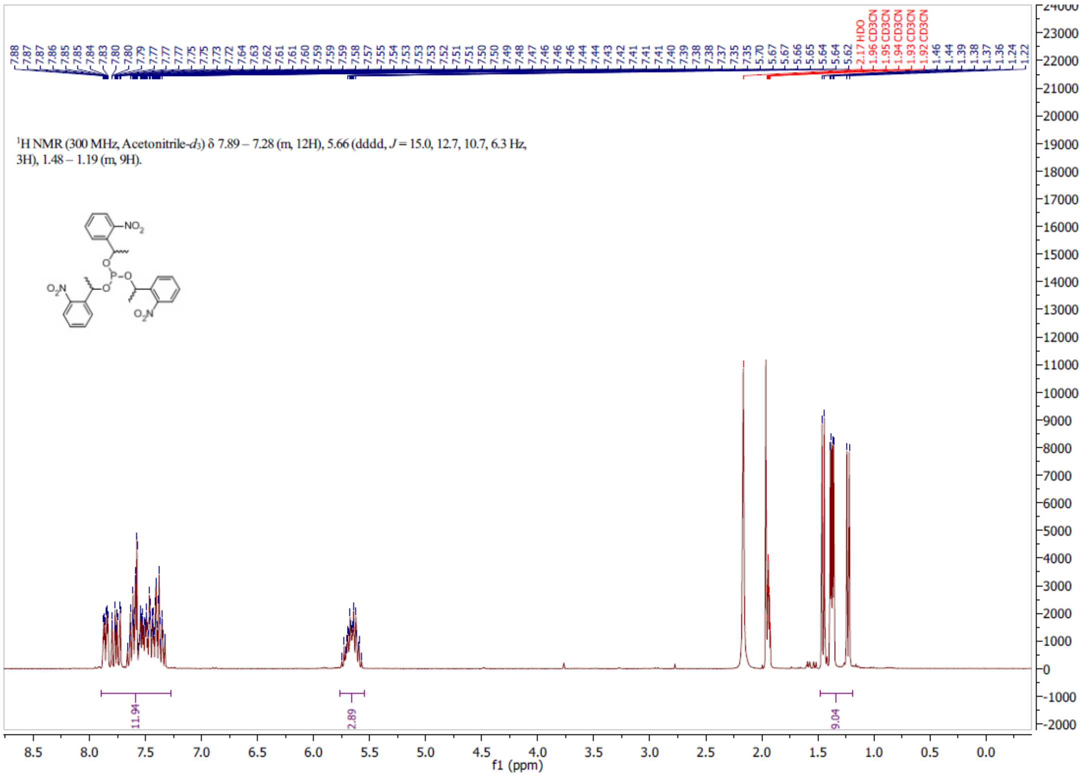

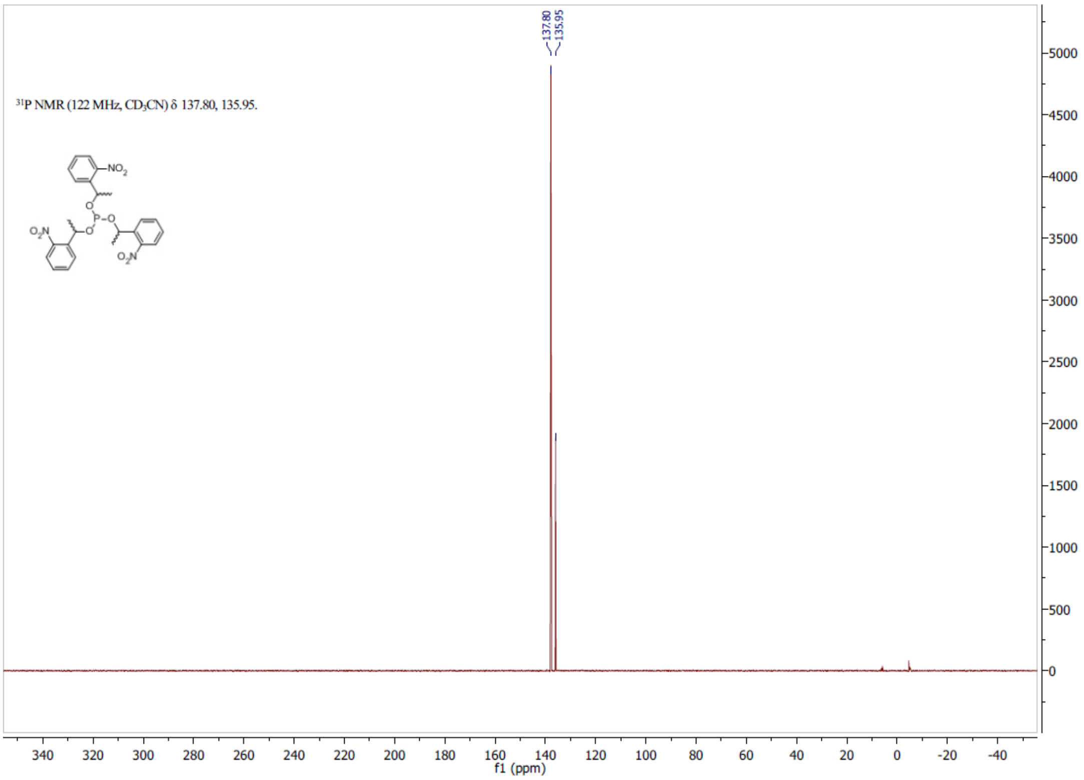
31P NMR (122 MHz, CD3CN) δ 137.80, 135.95.
137.80
135.95
NO2
O2N
O2N
f1 (ppm)

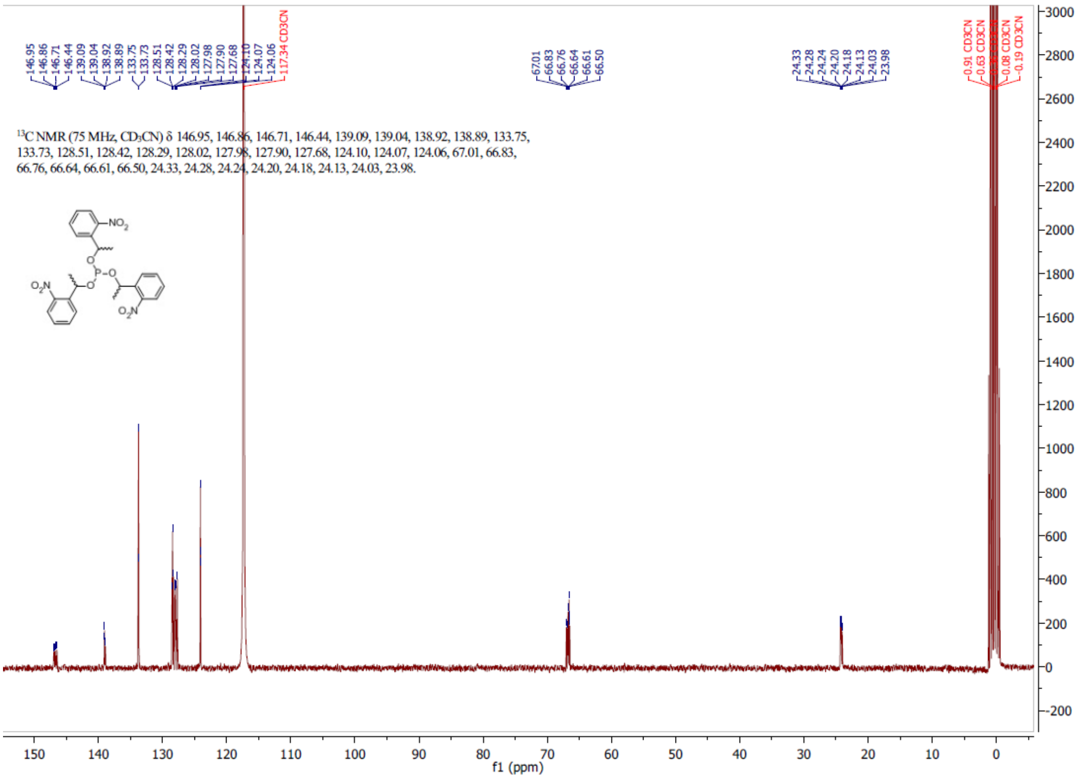
13C NMR (75 MHz, CD3CN) δ 146.95, 146.86, 146.71, 146.44, 139.09, 139.04, 138.92, 138.89, 133.75, 133.73, 128.51, 128.42, 128.29, 128.02, 127.98, 127.90, 127.68, 124.10, 124.07, 124.06, 67.01, 66.83, 66.76, 66.64, 66.61, 66.50, 24.33, 24.28, 24.24, 24.20, 24.18, 24.13, 24.03, 23.98.
117.34 CD3CN
0.91 CD3CN
0.63 CD3CN
0.08 CD3CN
-0.19 CD3CN
NO2
O2N
O2N
f1 (ppm)

6.References

1. Findeisen, M., T. Brand, and S. Berger, *A 1H-NMR thermometer suitable for cryoprobes.* Magnetic Resonance in Chemistry, 2007. **45**(2): p. 175-178.
2. Bax, A. and M.F. Summers, *Proton and carbon-13 assignments from sensitivity-enhanced detection of heteronuclear multiple-bond connectivity by 2D multiple quantum NMR.* Journal of the American Chemical Society, 1986. **108**(8): p. 2093-2094.
3. Cicero, D.O., G. Barbato, and R. Bazzo, *Sensitivity Enhancement of a Two-Dimensional Experiment for the Measurement of Heteronuclear Long-Range Coupling Constants, by a New Scheme of Coherence Selection by Gradients.* Journal of Magnetic Resonance, 2001. **148**(1): p. 209-213.
4. Piotto, M., V. Saudek, and V. Sklenář, *Gradient-tailored excitation for single-quantum NMR spectroscopy of aqueous solutions.* Journal of Biomolecular NMR, 1992. **2**(6): p. 661-665.
5. Webb, M.R., *A continuous spectrophotometric assay for inorganic phosphate and for measuring phosphate release kinetics in biological systems.* Proceedings of the National Academy of Sciences, 1992. **89**(11): p. 4884-4887.
6. Becke, A.D., *Density‐functional thermochemistry. III. The role of exact exchange.* The Journal of Chemical Physics, 1993. **98**(7): p. 5648-5652.
7. Lee, C., W. Yang, and R.G. Parr, *Development of the Colle-Salvetti correlation-energy formula into a functional of the electron density.* Physical Review B, 1988. **37**(2): p. 785-789.
8. Vosko, S.H., L. Wilk, and M. Nusair, *Accurate spin-dependent electron liquid correlation energies for local spin density calculations: a critical analysis.* Canadian Journal of Physics, 1980. **58**(8): p. 1200-1211.
9. Weigend, F., *Accurate Coulomb-fitting basis sets for H to Rn.* Physical Chemistry Chemical Physics, 2006. **8**(9): p. 1057-1065.
10. Weigend, F. and R. Ahlrichs, *Balanced basis sets of split valence, triple zeta valence and quadruple zeta valence quality for H to Rn: Design and assessment of accuracy.* Physical Chemistry Chemical Physics, 2005. **7**(18): p. 3297-3305.
11. Balasubramani, S.G., et al., *TURBOMOLE: Modular program suite for ab initio quantum-chemical and condensed-matter simulations.* The Journal of Chemical Physics, 2020. **152**(18): p. 184107.
12. Treutler, O. and R. Ahlrichs, *Efficient molecular numerical integration schemes.* The Journal of Chemical Physics, 1995. **102**(1): p. 346-354.
13. Eichkorn, K., et al., *Auxiliary basis sets for main row atoms and transition metals and their use to approximate Coulomb potentials.* Theor. chem. acc. 97 (1997) S. 119-124., 1997.
14. Schaftenaar, G., E. Vlieg, and G. Vriend, *Molden 2.0: quantum chemistry meets proteins.* Journal of Computer-Aided Molecular Design, 2017. **31**(9): p. 789-800.
15. Schaftenaar, G. and J.H. Noordik, *Molden: a pre- and post-processing program for molecular and electronic structures*.* Journal of Computer-Aided Molecular Design, 2000. **14**(2): p. 123-134.
16. Petrillo, D.E., et al., *A General Preparation of Protected Phosphoamino Acids.* Organic Letters, 2012. **14**(5): p. 1206-1209.
17. Hofmann, F.T., et al., *A phosphoarginine containing peptide as an artificial SH2 ligand.* Chemical Communications, 2011. **47**(37): p. 10335-10337.
